# E3 ligase RNF5 inhibits type I interferon response in herpes simplex virus keratitis through the STING/IRF3 signaling pathway

**DOI:** 10.3389/fmicb.2022.944101

**Published:** 2022-08-02

**Authors:** Zhi Liu, Likun Xia

**Affiliations:** Department of Ophthalmology, Shengjing Hospital of China Medical University, Shenyang, China

**Keywords:** HSV-1, herpes simplex keratitis, innate immunity, type I interferon, RNF5, STING

## Abstract

Herpes simplex keratitis (HSK), caused by the herpes simplex virus 1 (HSV-1), is a major blinding disease in developed countries. HSV-1 can remain latent in the host for life and cannot be eradicated. The infection causes the secretion of various cytokines and aggregation of inflammatory cells. In the early stage of inflammation, mainly neutrophils infiltrate the cornea, and CD4^+^ T cells mediate the immunopathological changes in herpetic stromal keratitis in the subsequent progression. The STING/IRF3-mediated type I interferon (IFN) response can effectively inhibit viral replication and control infection, but the activity of STING is affected by various ubiquitination modifications. In this study, we found that the expression of RNF5 was elevated in corneal tissues and corneal epithelial cells after infection with HSV-1. Immunofluorescence staining confirmed that RNF5 was mainly expressed in the corneal epithelial layer. We silenced and overexpressed RNF5 expression in corneal epithelial cells and then inoculated them with HSV-1. We found that the expressions of STING, p-IRF3, p-TBK1, and IFN-β mRNA increased after RNF5 silencing. The opposite results were obtained after RNF5 overexpression. We also used siRNA to silence RNF5 in the mouse cornea and then established the HSK model. Compared with the siRNA-control group, the siRNA-RNF5 group showed significantly improved corneal inflammation, reduced clinical scores and tear virus titers, and significantly increased corneal IFN-β expression. In addition, the expressions of the proinflammatory cytokines IL-6 and TNF-α in the corneal tissue were significantly decreased, indicating that RNF5 silencing could effectively promote IFN-I expression, inhibit virus replication, alleviate inflammation, and reduce corneal inflammatory damage. In summary, our results suggest that RNF5 limits the type I IFN antiviral response in HSV corneal epithelitis by inhibiting STING/IRF3 signaling.

## Introduction

Herpes simplex keratitis (HSK), which is caused by herpes simplex virus 1 (HSV-1) infection, is the main cause of infectious blindness in developed countries (Farooq and Shukla, 2012). According to epidemiological statistics, 50–90% of people worldwide carry HSV-1, and the HSV seroprevalence rate among American adults is as high as 90% (Liesegang, 2001). HSV-1 can infect any part of the eye, but most commonly manifests as epithelial keratitis or dendritic keratitis (Lobo et al., 2019). The virus gains entry through direct contact with the mucosa. At this initial stage, symptoms caused by infection are milder. After the initial stage, HSV-1 enters the trigeminal ganglion through the corneal nerve and can continue to replicate in the trigeminal ganglion or enter a latent state. Under the influence of stress, fever, ultraviolet radiation, weakened immunity, and other factors, the virus is reactivated, leading to recurrent infections (Poccardi et al., 2019). Although HSV corneal epithelitis resolves spontaneously within a week, without proper treatment, it could trigger the development of HSK and immune-mediated infiltration of the stroma, resulting in progressive corneal opacity and neovascularization that directly affects vision (Lobo et al., 2019). The earliest sign of HSK disease is neutrophil infiltration (Daheshia et al., 1998). In subsequent secondary or recurrent infection, a larger neutrophil infiltration occurs and is clinically evident (Osorio et al., 2002). As discovered by the study of a severe combined immunodeficiency mouse model, after HSV-1 infects the cornea, it causes neutrophils to aggregate to help clear the virus and act as an agonist to perpetuate the CD4^+^ T cell-mediated inflammatory response (Thomas et al., 1997). Studies have shown that CD4^+^ T cells mediate the immunopathological changes in herpetic stromal keratitis, and ocular lymphocytes were identified as Th1 cells (Niemialtowski and Rouse, 1992a,b).

During HSV-1 infection, inflammation and angiogenesis trigger each other, cause corneal opacity and haze, and can ultimately result in blindness (Azar, 2006). HSV-1 directly induces corneal vascularization by upregulating VEGF-A expression (Wuest and Carr, 2010). Silencing miR-132 can affect the production of VEGF-A and IL-17 and thus effectively inhibit the formation of HSK neovascularization (Mulik et al., 2012). Knockout of miR-155 significantly suppressed type 1 and 17 responses of T helper cells in ocular and lymphoid organs and relieved the degree of corneal stromal lesions (Bhela et al., 2015).

Topical GCV is currently the first choice for the treatment of HSK (Tsatsos et al., 2016). However, the use of antiviral drugs alone cannot effectively control the corneal opacity caused by the infiltration of a large number of inflammatory cells. Therefore, there is an urgent need to clarify the specific mechanism of HSK to formulate exact and effective prevention and treatment plans.

The innate immune response is the host’s first line of defense against invading pathogens. Pathogen invasion triggers the innate immune response, which initiates the secretion of a series of cytokines and activates immune cells to limit the pathogens and maintain the immune balance of the body (Wu and Chen, 2014). After HSV-1 invades cells, viral DNA and its products are recognized by intracellular sensors and promote antiviral responses through the secretion of proinflammatory cytokines and type I interferons (IFNs), thereby inhibiting viral replication and controlling the infection (Xia et al., 2016). Once the DNA is recognized, the signal is converged to a common adaptor protein, the stimulator of IFN genes (STING), which promotes the phosphorylation of IRF3 through TANK-binding kinase 1 (TBK1). Phosphorylated IRF3 forms p-IRF3 dimers and translocates to the nucleus, inducing transcription of interferon-stimulated genes (ISGs), which in turn promotes IFN-β expression (Phelan et al., 2020). Mice lacking STING exhibit an impaired response to HSV-1 infection and decreased cytoplasmic DNA stimulation and type I IFN production (Ni et al., 2018; Dogrammatzis et al., 2020). Thus, STING, as a central adaptor protein of cytoplasmic DNA sensors, plays an indispensable role in activating type I IFN signaling after DNA viruses invade host cells (Wan et al., 2020).

RNF proteins are a group of transmembrane proteins containing a unique three-dimensional domain consisting of C3HC4 amino acid residues and eight conserved cysteine and histidine residues that bind two zinc cations (Cham et al., 2018). The RING domain is an important factor that enables the members of the RNF family to function as ubiquitin ligases (Zheng and Gao, 2019). Transmembrane RNF proteins play important roles in many organelles and cellular processes, including protein transport (Amal et al., 2019); cell proliferation, differentiation, and apoptosis (Wei et al., 2019); immune regulation (Geng et al., 2017); and mitochondrial dynamics (Adir et al., 2021). Studies have shown that the E3 ubiquitin ligase RNF5 interacts with STING in a viral infection-dependent manner in cells infected with an RNA virus, vesicular stomatitis virus (VSV; Zhong et al., 2009).

Although the host initiates a series of antiviral immune-inflammatory responses, it is still unable to completely remove the herpes virus from the body. In the early stage of viral invasion of corneal cells, the viral gene products can modify various host proteins and participate in the host’s ubiquitin-proteasome system, thus avoiding recognition by the host immune system (Sun et al., 2017, 2021; Rodríguez et al., 2020). Therefore, we aimed to determine whether the host-derived E3 ubiquitin ligase RNF5 is involved in the infection process of HSV-1 and characterize the role of RNF5 in the occurrence and development of HSK.

## Materials and methods

### Animals

Female BALB/c mice (age, 6–8 weeks; weight, 18–22 g each) were purchased from Skbex Biotechnology Company (Henan, China). The mice were raised in a specific pathogen-free environment with suitable temperature and humidity, under a 12-h light and 12-h dark environment, and given sufficient water and food. All investigations followed the guidelines of the Institutional Animal Care and Use Committee of China Medical University and adhered to the ARVO Statement for the Use of Animals in Ophthalmic and Vision Research. All procedures were performed with the animals under isoflurane anesthesia, and only one cornea of each mouse was infected.

### Cells

Human corneal epithelial cells (ZQ1003) were purchased from Shanghai Zhong Qiao Xin Zhou Biotechnology Co., Ltd. (Shanghai, China). The cells were grown in Dulbecco-modified Eagle medium (DMEM)/nutrient mixture F-12 supplemented with 10% fetal bovine serum (FBS; Hyclone, UT, United States), 1% penicillin-streptomycin solution (100×), 5 UG/ml recombinant human insulin, and 10 ng/ml recombinant human epidermal growth factor. Vero cells (ATCCCCL81) were maintained in DMEM supplemented with 10% FBS and cultured at 37°C with 5% CO_2_ in an environment with sufficient humidity.

### Virus

The virus used in this study was the McKrae strain (10^6.8^ TCID_50_/ml), which is a human isolate of HSV-1. The virus was recovered and proliferated on a well-grown monolayer of Vero cells, cultured in DMEM containing 5% FBS. After virus harvesting, the extracted virus-containing liquid was stored at −80°C until the subsequent experiments.

### Animal model

Mice were acclimated to the environment for 1 week after arriving in the laboratory, and levofloxacin eye drops were instilled in both eyes daily for 3 days before the establishment of the animal model to prevent infection by other pathogens. After inducing isoflurane anesthesia, we scratched the character “#” into the corneal epithelium with a 1-ml syringe. Then, 5 μl of the virus solution was added dropwise over the scratched corneal surface; in the control group, 5 μl DMEM was added dropwise. The eyelid was then closed and massaged gently to facilitate contact of the virus solution (or control medium) with the cornea. The corneal lesions of the mice were observed under a microscope every day. The severity of the eye infection was scored using a 6-point scale in a blinded fashion (Chen et al., 2021): 0 points, intact epithelium (no lesion); 1 point, diffuse punctate lesions; 2 points, dendritic lesion occupying less than one-fourth of the entire epithelial area; 3 points, severe dendritic lesion extending across more than one-fourth of the entire epithelial area; 4 points, geographic lesion on the corneal epithelium; and 5 points, eye completely swollen shut.

### Tear virus titer assay

A swab dipped in sterile DMEM was used to gently collect tears from the corneal surface. The swab was then inserted into an Eppendorf tube containing 1 ml of sterile DMEM and mixed well. The tube was frozen at −80°C for later use. The tears were diluted in a 10-fold gradient, added to a monolayer of Vero cells, and cultured for 48 h. The virus titer was then calculated.

### Quantitative polymerase chain reaction analysis

RNA isolater Total RNA Extraction Reagent (R401-01, Vazyme, Nanjing, China) was used to extract total RNA from the samples. Total mRNA was reverse transcribed using the PrimeScript™ RT reagent kit with gDNA Eraser to generate cDNA (Takara, Japan). Quantitative PCR was performed using SYBR Green (Takara, Japan) and a 7500 Fast Real-Time PCR detection system (Applied Biosystems, CA, United States). The sequences of the quantitative PCR primers are shown in Table 1.

**TABLE 1:** qPCR primers.

Gene	Species	Forward primer sequence [5′–3′]	Reverse primer sequence [5′–3′]
RNF5	Human	GAGGTGGTTTCTGGTTTGTTGG	ATGGCCGGTTTTGTTTCGCC
RNF5	Mouse	CTCCTTTGGTGTCGGTGCCTTC	GCGAGGAACAGGAATAGGGAATCTTG
IFN-β	Human	GGACGCCGCATTGACCATCTATG	TCAACAATAGTCTCATTCCAGCCAGTG
IFN-β	Mouse	TGGGTGGAATGAGACTATTGTTGTACG	CAAGTGGAGAGCAGTTGAGGACATC
GAPDH	Human	CAGGAGGCATTGCTGATGAT	GAAGGCTGGGGCTCATTT
GAPDH	Mouse	GTTGTCTCCTGCGACTTCA	TGGTCCAGGGTTTCTTACTCC

### Western blotting

The corneal samples and HCECs were lysed using RIPA buffer (EpiZyme, Shanghai, China), and total proteins in the supernatants were quantified using a BCA protein assay kit (EpiZyme, Shanghai, China). The total protein from the mouse corneas (3 corneas/sample/group) and HCECs was extracted and prepared in a standardized manner for Western blotting. After sodium dodecyl sulfate-polyacrylamide gel electrophoresis, the proteins were transferred to polyvinylidene fluoride membranes. Next, the membranes were blocked with 5% non-fat milk at room temperature for 2 h and incubated overnight at 4°C with the following primary antibodies: anti-RNF5 antibody (1:1,000; Abcam, United Kingdom), STING (D2P2F) rabbit mAb (1:1,000; Cell Signaling Technology), IRF-3 (D83B9) rabbit mAb (1:1,000; Cell Signaling Technology), phospho-IRF3 (D6O1M) rabbit mAb (1:1,000; Cell Signaling Technology), TBK1 (D1B4) rabbit mAb (1:1,000; Cell Signaling Technology), phospho-TBK1 (D52C2) rabbit mAb (1:1,000; Cell Signaling Technology), TNF-α antibody (1:1,000; Cell Signaling Technology), IL-6 antibody (abs135607, 1:1,000; Absin, Shanghai, China), and GAPDH antibody (P60036, 1:3,000; Abmart, Shanghai, China). After the incubation, the membranes were washed 3 times with Tris-buffered saline. Then, the membranes were incubated with the secondary antibody goat anti-rabbit IgG (H + L) (peroxidase/horseradish peroxidase-conjugated, 1:5,000; Elabscience, Wuhan, China) for 2 h at room temperature. Finally, an enhanced chemiluminescence kit (Wanlei, Shenynag, China) was used to visualize the membranes. Densitometry analysis was performed using the ImageJ 6.0 software.

### Histopathology and immunofluorescence staining

The mouse eyeballs were harvested at the appropriate experimental time points, rinsed with phosphate-buffered saline (PBS), and fixed with 4% paraformaldehyde for 24 h. Next, the eyeball tissue was dehydrated, fixed, embedded in paraffin, and sectioned at 4-μm intervals. The paraffin sections were used for hematoxylin and eosin staining. For the immunofluorescence experiments, the paraffin sections were deparaffinized, subjected to antigen heat retrieval, and then blocked with 20% goat serum in a blocking buffer for 1 h. The sections were soaked in the primary antibody anti-RNF5 antibody (Abcam, United Kingdom) and incubated overnight at 4°C. After three 5-min washes in the wash buffer (PBS), the sections were incubated with the fluorescent goat anti-rabbit IgG antibody (Boster, Wuhan, China) for 30 min at 37°C. After another three 5-min washes with the wash buffer, the sections were incubated with DAPI solution (Solarbio, Beijing, China) for 5 min at room temperature. After three more 5-min washes with the wash buffer, the slides were mounted using an anti-fluorescence quenching mounter (Seven, Beijing, China). The cells used for fluorescent staining were fixed with 4% paraformaldehyde for 30 min, and the rest of the steps were the same as those used for the tissue sections. Both the cells and tissues were observed under a fluorescence microscope (Eclipse NI, Nikon, Japan).

### siRNA and plasmids

The siRNAs used in this experiment were purchased from Guangzhou Ribobio Co., Ltd. (Guangzhou, China), and the plasmids used in this experiment were purchased from Shanghai Genechem Co., Ltd. (Shanghai, China). The siRNAs were administered to the animals at a dose of 3 nmol/animal/day, by subconjunctival injection, for 3 consecutive injections before the virus inoculation. The dosages of the siRNAs and plasmids were determined in a pre-experiment. The proximal sequence element of the plasmids was CMV enhancer-RNF5 (NM_006913)-SV40-puromycin.

### Statistical analysis

All experiments were performed three times. Data were expressed as mean ± SD. Differences between the experimental groups were analyzed using the Student *t*-test. A *p*-value of <0.05 was considered statistically significant. The GraphPad Prism software (GraphPad Software Inc., La Jolla, CA, United States) was used for statistical analysis. All methods were performed following the relevant guidelines and regulations.

## Results

### Corneal RNF5 expression is increased in herpes simplex keratitis mice

**FIGURE 1:**
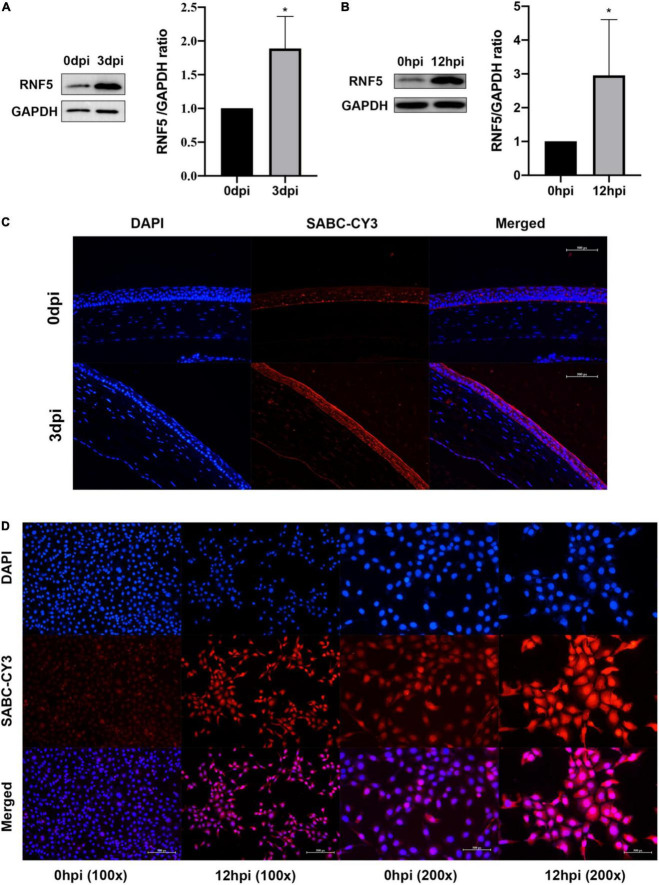
RNF5 is highly expressed after HSV-1 infection. **(A)** The mouse cornea was inoculated with HSV-1 to establish an HSK model. The corneas were harvested for Western blot analysis on days 0 and 3 after the infection. Three corneas were tested in each group. The data are shown as the mean ± SD of three independent experiments. *P < 0.05. **(B)** Human corneal epithelial cells (HCECs) were inoculated with HSV-1 at a multiplicity of infection (MOI) of 10. At 12 h after the infection, the expression of RNF5 was detected using Western blotting (mean ± SD). *P < 0.05. **(C)** On days 0 and 3 after the infection, the expression of RNF5 in the cornea was assessed using immunofluorescence. The blue fluorescence indicates the nucleus, and the red fluorescence indicates RNF5 expression (magnification, 200×). **(D)** Immunofluorescence staining was performed 12 h after the HCECs were infected with HSV-1. The nucleus appears blue, and RNF5 expression appears red (magnification, 100× and 200×).

We established an HSV-1 infection model by using BALB/c mice. On the third day after the infection, a microscopic examination of the cornea revealed disordered epithelial cells, abundant inflammatory-cell infiltration, and inflammation of mainly the corneal epithelial layer, which proved that the model had been successfully established. Total proteins were extracted from the corneal tissue and analyzed using Western blotting. The results showed that the expression level of RNF5 was significantly increased compared with the baseline level (on the day of infection) (Figure 1A). Immunofluorescence staining of the cornea revealed that the intensity of the fluorescence staining was stronger at 3 days after the infection than at the baseline (Figure 1C). The increase in the staining intensity is statistically significant, *P* < 0.05.

### RNF5 is highly expressed in human corneal epithelial cells inoculated with herpes simplex virus 1

We also established a model of human corneal epithelial cell (HCEC) infection with HSV-1 and collected the cells at 12 h after the infection for immunofluorescence (Figure 1D) and Western blotting analyses (Figure 1B). The results showed that the expression of RNF5 in the cells was significantly increased after infection with HSV-1. RNF5 degrades STING through K48 ubiquitination and affects cellular anti-RNA virus immune responses (Zhong et al., 2010). Therefore, we next explored the role and specific molecular mechanisms of RNF5 in infection with the double-stranded DNA virus HSV-1.

### RNF5 silencing and overexpression in human corneal epithelial cells

**FIGURE 2:**
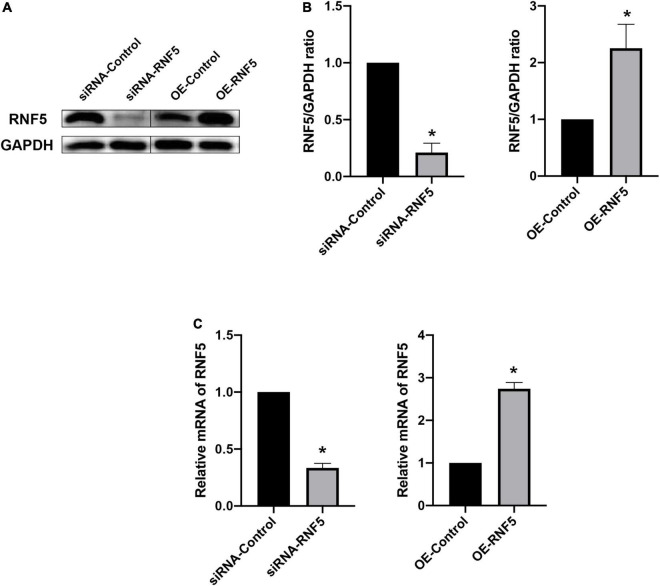
Silencing and overexpression of RNF5 in HCECs. **(A)** At 24 h after the transfection of siRNAs and plasmids, the expression of RNF5 in HCECs was detected using Western blotting. **(B)** Relative gray value statistics, with GAPDH as the internal reference. The left side is the silenced group, and the right side is the overexpression group. The transfection efficiency was as expected. **(C)** At 24 h after the transfection of siRNAs and plasmids, the expression of RNF5 mRNA was detected, with GAPDH as the internal reference. Data are shown as mean ± SD of three independent experiments. *P < 0.05.

To clarify the role of RNF5 in the process of HCEC infection with HSV-1, we constructed small interfering (siRNA)-RNF5 and RNF5 overexpression plasmids, along with the corresponding siRNA-control and negative control plasmids. The cells were transfected with Lipo3000 (GlpBio, Montclair, CA, United States) and harvested 24 h after transfection. The total RNA and total protein were extracted, and a polymerase chain reaction (PCR) assay (Figure 2C) and Western blot analysis (Figures 2A,B) were performed to determine the efficiency of silencing and overexpression.

### RNF5 inhibits STING/IRF3 signaling after herpes simplex virus 1 infection

**FIGURE 3:**
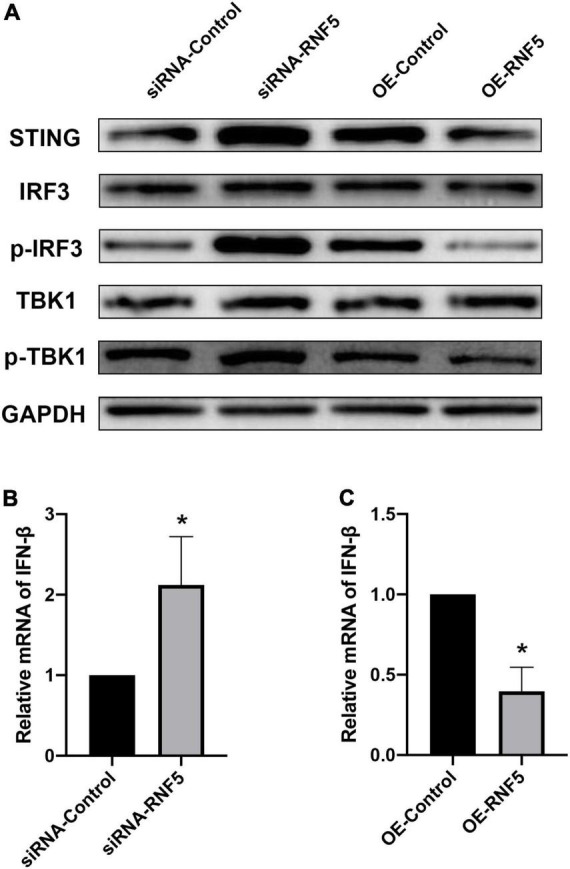
RNF5 inhibits the expression of IFN-β through the STING/IRF3 signaling pathway. **(A)** HCECs were inoculated with HSV-1 after the silencing or overexpression of RNF5, and the relative expression levels of STING, IRF3, p-IRF3, TBK1, and p-TBK1 were detected using Western blotting at 12 h after the infection. **(B)** HCECs were inoculated with HSV-1 after RNF5 silencing, and 12 h after the infection, the expression of IFN-β mRNA was detected using quantitative PCR. **(C)** HCECs were inoculated with HSV-1 after inducing RNF5 overexpression, and the expression of IFN-β mRNA was detected using quantitative PCR at 12 h after the infection. Data are shown as the mean ± SD of three independent experiments. *P < 0.05.

After HSV-1 enters cells, it is recognized by pattern-recognition receptors, which activate the innate immune defense. Among the defense responses, the cGAS/STING signaling pathway plays an important role. Activated STING can recruit TBK1 and IRF3 to form a complex that promotes IRF3 phosphorylation and dimerization and then the dimerized p-IRF3 enters the nucleus to activate ISGs, which can promote type I IFN gene transcription (You et al., 2019; Phelan et al., 2020). To explore the molecular mechanism of the effects of RNF5 on the type I IFN response in HSK, we transfected HCECs with siRNA-RNF5, siRNA-control, RNF5 overexpression plasmid, and negative control plasmid, respectively. HSV-1 was inoculated at a multiplicity of infection of 10 at 24 h after the transfection. The PCR results showed that RNF5 silencing was followed by significantly increased IFN-β expression during HSV-1 infection (Figure 3B), whereas RNF5 overexpression was associated with inhibition of IFN-β expression (Figure 3C). When RNF5 was silenced, STING ubiquitination and degradation were attenuated. Compared with the siRNA-control group, the RNF5-siRNA group showed increased STING expression, increased recruitment, and activation of IRF3 and TBK1, and increased p-IRF3 and p-TBK1 contents (Figure 3A). It has been reported that in Sendai virus and vesicular stomatitis virus infections, RNF5 mediates the K48 ubiquitination and degradation of IRF3 (Zhang et al., 2021), which is consistent with our observation of increased IRF3 expression after RNF5 silencing. The above results were verified by analyzing the RNF5-overexpressing HCECs. Western blotting showed that compared with the negative control group, the RNF5 plasmid overexpression group showed significantly reduced contents of STING, IRF3, p-IRF3, and p-TBK1 (Figure 3A). Collectively, these results indicate that during HCEC infection with HSV-1, RNF5 could aggravate the infection by inhibiting the STING/IRF3 signaling pathway and reducing the expression of IFN-β.

### Silencing of RNF5 in corneal tissue

**FIGURE 4:**
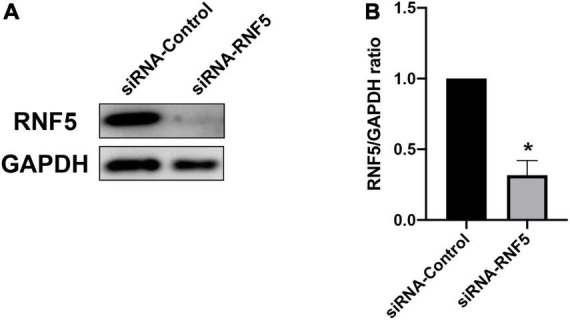
Silencing of RNF5 expression in corneal tissue. **(A)** siRNA pretreatment was used to silence the expression of RNF5 in corneal tissue. The mouse corneas (3 corneas/group) were harvested on the third day after the injection to detect the expression of RNF5. **(B)** Relative gray value statistics, with GAPDH as the internal control, show that RNF5 was effectively silenced in the cornea. Data are shown as the mean ± SD of three independent experiments. *P < 0.05.

The results of the cellular experiments indicate that RNF5 can limit the antiviral effect of the type I IFN response. Therefore, we attempted to analyze the *in vivo* effects of RNF5 silencing. The right eyes of the mice were injected subconjunctivally with siRNA-RNF5 every day for 3 consecutive days (3 nmol/mouse). The mice in the control group were injected with the same amount of siRNA control. Appropriate injection methods and doses had been confirmed in preliminary experiments. On the third day after the injection, the total RNA and total protein of the mouse cornea were extracted for PCR assays (Figure 4B) and Western blot analysis (Figure 4A). The results show that the silencing efficiency was as expected.

### Corneal RNF5 silencing attenuates the severity of herpes simplex keratitis

Mice were injected subconjunctivally with siRNA for 3 consecutive days to silence the localized RNF5 expression in the cornea. The HSV-1 solution was then inoculated into the right eyes of the mice. On the third day after the infection, total RNA and total protein were extracted from the mouse corneal tissue and were subjected to PCR assays and Western blot analysis.

**FIGURE 5:**
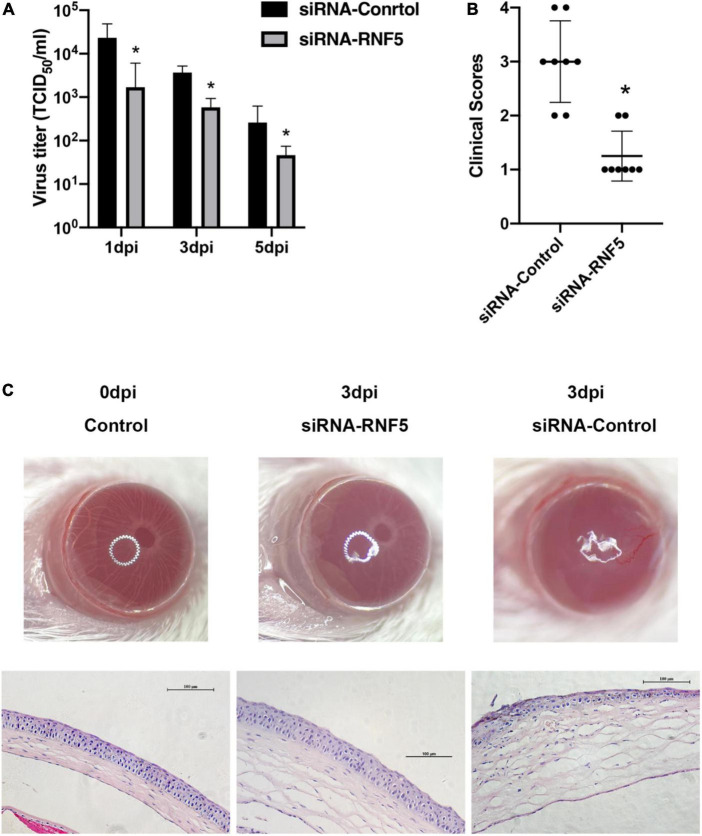
Silencing RNF5 alleviates HSK severity in mice. **(A)** Effects of RNF5 silencing on viral titers in tears at 1, 3, and 5 days after HSV-1 infection in mice. **(B)** Clinical scores of mice infected with HSV-1 on day 3 after RNF5 silencing (8 mice/group). **(C)** Photographs showing the corneal appearance and hematoxylin and eosin staining of the corneal tissue in mice infected with HSV-1 after RNF5 silencing (magnification, 200×). *P < 0.05.

**FIGURE 6:**
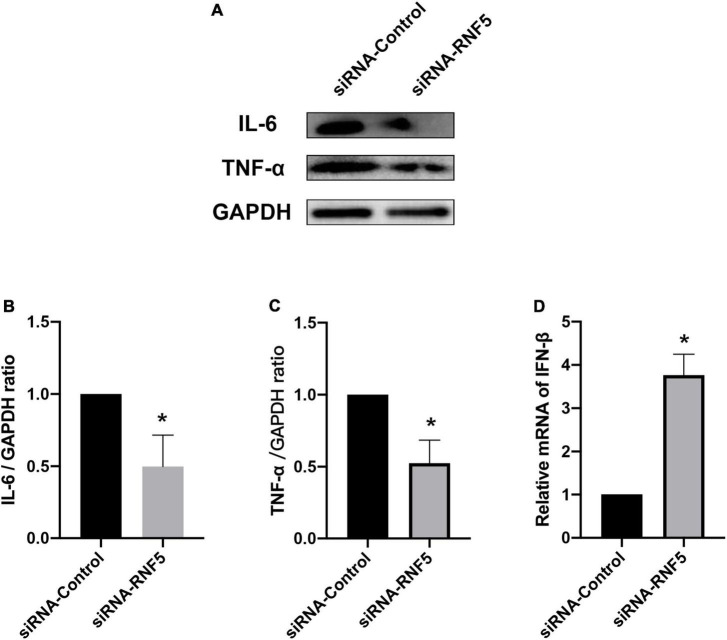
Effects of RNF5 silencing on cytokine secretion in mouse HSK. **(A)** Mice were infected with HSV-1 after RNF5 silencing, and the mouse corneas (3 mice/group) were harvested on the 3rd day after the infection. The expression levels of interleukin (IL)-6 and tumor necrosis factor (TNF)-α were detected by Western blotting. **(B)** Statistical graph of relative gray values of corneal IL-6 levels, with GAPDH as the internal reference. **(C)** Statistical graph of relative gray values of corneal TNF-α levels, with GAPDH as the internal reference. **(D)** Mice were infected with HSV-1 after RNF5 silencing, and the mouse corneas (3 mice/group) were harvested on the 3rd day after the infection. The mRNA expression of RNF5 was detected using quantitative PCR, with GAPDH as the internal reference. Data are shown as mean ± SD of three independent experiments. *P < 0.05.

The experimental results showed that compared with the siRNA-control group, the mice in the siRNA-RNF5 group had less corneal opacity, fewer ulcers, and a lower average clinical score (Figure 5B). Pathological examination (Figure 5C) showed that in the uninfected control group, the corneal epithelium was arranged regularly, and the thickness of the stromal layer was appropriate without any obvious hyperplasia. In the siRNA-control group, the corneal epithelium was disordered; the stromal layer was thickened; the collagen fibers were disordered; numerous neutrophils infiltrated the cornea; and the inflammation involved the stromal layer. In the siRNA-RNF5 group, the cells of the corneal epithelial layer were slightly disordered, and the number of inflammatory cells was significantly lower than that in the siRNA-control group. The tear analysis showed that the virus titers of the tears were lower in the RNF5-siRNA group than in the siRNA-control group at 1, 3, and 5 days after the infection (Figure 5A). The PCR results showed that the expression of IFN-β in the corneal tissue in the RNF5-siRNA group was three times that in the siRNA-control group (Figure 6D). The increased expression of IFN-β effectively restricted viral replication and exerted a highly efficient antiviral effect. It also inhibited the excessive secretion of proinflammatory cytokines. Therefore, we next analyzed the contents of proinflammatory factors in the corneal tissue by Western blotting (Figure 6A). The results showed that the expression levels of IL-6 (Figure 6B) and TNF-α (Figure 6C) in the cornea were significantly higher in the siRNA-control group than in the siRNA-RNF5 group. These results indicate that RNF5 silencing can promote the antiviral effect of the type I IFN response and reduce the severity of HSK. These findings confirm that RNF5 plays a key role in the occurrence and development of HSK.

## Discussion

After HSV-1 infects the cornea, its DNA is recognized by the cytosolic DNA sensors. Common DNA sensors include cyclic GMP-AMP synthase (cGAS; Bode et al., 2016), DNA-dependent activator of interferon (IFN)-regulatory factors (IRFs; Zhang et al., 2016), DEAD-box polypeptide 41 (Ma et al., 2018), and IFN-γ-inducible protein 16 (IFI16; Diner et al., 2015; Coulon et al., 2019). After the activation of type I IFN signaling, the expression of multiple IFN-stimulated genes (ISGs) is induced through positive feedback regulation, thereby enhancing the innate immune response. The final product of these signaling cascades is IFN-α/β, which binds to type I IFN receptors in an autocrine and paracrine manner, and upon receptor stimulation, activates downstream antiviral pathways such as the oligoadenylate synthetase, protein kinase R, and RNase L pathways (Christensen et al., 2016). These pathways inhibit further viral replication in host cells.

Protein degradation mediated by the ubiquitin-proteasome pathway is an important mechanism that regulates the levels and functions of intracellular proteins. The E3 ubiquitin ligase labels target substrates by linking the lysine residues of the substrate with ubiquitin molecules. Polyubiquitination, which occurs on the lysine residues K29 and K48, causes mainly proteasomal degradation of the target substrate, whereas polyubiquitination at other sites (K6, K11, K27, K63, and M1) and monoubiquitination are involved in the regulation of inflammation, innate immunity, endocytic transport, transcriptional regulation, and DNA repair (Pickart, 2001; Husnjak and Dikic, 2012; Zheng and Gao, 2019). The activity of STING is regulated by various ubiquitin modifications. For example, the E3 ubiquitin ligases TRIM56 and TRIM32 catalyze the K63-linked ubiquitination of STING upon viral infection, thereby promoting STING recruitment and STING dimerization by TBK1 (Zhang et al., 2012). TRIM29 catalyzes the K48 ubiquitination of STING and degrades it to inhibit the anti-HSV-1 type I IFN response (Li et al., 2018). Viral infection induces the E3 ligase AMFR/gp78 to constitutively interact with STING and mediate the K27-linked ubiquitination of STING, which is critical for the STING-mediated recruitment of TBK1 and IRF3 following DNA virus infection (Wang et al., 2014). Studies have shown that RING finger 5 (RNF5) targets STING for K48-linked ubiquitination and proteasome-dependent degradation during infection by the RNA virus Sendai virus, thereby inhibiting the activation of downstream signaling pathways (Zhong et al., 2010).

Growing data have revealed the underlying molecular mechanisms of virus–host confrontation in the innate immune response (Soh et al., 2022). HSV-1 tegument protein VP22 inhibits AIM2-dependent inflammasome activation and IL-1b secretion in infected macrophages (Maruzuru et al., 2018); in addition, HSV-1 VP22 inhibits the enzymatic activity of cGAS, thereby inhibiting cGAS/STING-mediated antiviral signaling (Huang et al., 2018). It is reported that HSV-1-infected cell proteins ICP0, ICP27, and ICP34.5 are involved in interfering with type I IFN signaling (Mossman et al., 2000; Verpooten et al., 2009; Tian et al., 2013). ICP27 interacts with the activated STING–TBK1 signalosome to inhibit the phosphorylation and activation of IRF3, thereby limiting the STING–TBK1–IRF3 pathway (Christensen et al., 2016). HSV-1 synthesizes the E3 ubiquitin ligase-infected cell polypeptide 0 (ICP0), which inhibits and terminates tumor necrosis factor (TNF)-α and Toll-like receptor (TLR)-mediated NF-κB activation (Daubeuf et al., 2009) and also degrades IFI16 to block DNA sensing (Lanfranca et al., 2014); these changes suppress the host-cell innate immunity and thereby promote viral infection. HSV-1 also encodes the deubiquitinase UL36, which deubiquitinates TRAF3 to hinder the recruitment of TBK1 (Wang et al., 2013). It was found that RNF90 enhances the K48-linked ubiquitination of STING and thereby degrades it, which inhibits the host’s innate immune response to HSV-1 (Yang et al., 2020).

RNF5 is a membrane-anchored E3 ubiquitin ligase involved in ER-associated protein degradation (Kuang et al., 2013). It controls the clearance of misfolded proteins, including mutated cystic fibrosis transmembrane regulator (CFTR) and glutamine carrier proteins, which are abnormally folded during chemotherapy-induced ER stress (Younger et al., 2006; Jeon et al., 2015). RNF5 also controls the stability of autophagy-related four B cysteine peptidase (ATG4B) proteins (Kuang et al., 2012). Hence, RNF5 can potentially impact immune-checkpoint control and tumor growth. For example, the changes in gut microbiota exhibited by Rnf5^–/–^ mice contribute to antitumor immunity and limit tumor expansion (Li et al., 2019). RNF5 functions in the early stages of F508del CFTR biosynthesis (Grove et al., 2011). RNF5 knockout was particularly effective in rescuing mutant CFTR at the functional level in immortalized bronchial epithelial cells (Sondo et al., 2018).

In one study, a co-immunoprecipitation assay showed that RNF5 mediates the polyubiquitination and degradation of viral protein 1 (VP1) of the foot-and-mouth disease virus (FMDV) by directly interacting with VP1. *In vitro* experiments have shown that RNF5 not only degrades FMDV VP1 but also degrades the VP1 of poliovirus, Seneca Valley virus, and enterovirus 71 and that these degradation processes depend on the E3 ligase activity of RNF5 (Sun et al., 2019). RNF5 shows broad-spectrum antiviral effects on some RNA viruses, which provide a theoretical basis for designing high-efficiency vaccine candidate strains (or antigens).

In the early stage of HSV-1 infection, the earliest antiviral cytokines secreted by the corneal tissue are type I IFNs (IFN-α and IFN-β), which play an important role in the early control of viral replication (Hendricks et al., 1991). The binding of IFNs to cell-surface receptors leads to multiple signaling cascades that ultimately generate multiple ISGs with antiviral properties. The normal transduction of type I IFN signaling is essential for the recruitment of immune cells to the site of infection (Leib et al., 1999). Although the pathogenesis of HSK is not fully understood, type I IFN response mediated by STING/IRF3 plays a major antiviral role in HSK. Another study has reported that RNF5 directly deubiquitinates IRF3 at position K48 (Zhang et al., 2021). Thus, RNF5 has an important inhibitory influence on the complex composed of STING, IRF3, and TBK1.

RNF5 is expressed in numerous tissues. Our study is the first to confirm that RNF5 is constitutively expressed in corneal tissues. In this study, the HSV-1 McKrae strain (10^6.8^ TCID_50_/ml) was inoculated into the right cornea of BALB/c mice after corneal scratching to establish the HSK epithelitis model. We detected a marked increase in RNF5 expression on the third day after the infection when the epithelitis was most obvious. Using immunofluorescence to detect the localization of RNF5 in the corneal tissue, we found that RNF5 was mainly expressed in the cytoplasm of the corneal epithelial cells. Moreover, the intensity of the fluorescence staining was significantly enhanced on the third day after the infection, which further confirmed the increased expression of RNF5 in the corneal tissue in HSK. We also established a model of HSV-1 infection of HCECs, and the results supported the above conclusion.

Next, we explored the effects of RNF5 on the STING/IRF3 signaling pathway at the cellular level. We used siRNA and plasmids to silence and overexpress RNF5, respectively, in HCECs to detect the effects of RNF5 on the STING/IRF3 signaling pathway. The results show that RNF5 silencing inhibited STING degradation, promoted the recruitment of IRF3 and TBK1 by STING, and increased the transcription of downstream IFN-β. RNF5 overexpression significantly reduced the STING content and thus inhibited STING/IRF3 signaling. These findings show that HSV-1 inhibits the antiviral effect of the type I IFN response by causing high RNF5 expression after entering cells, thereby aggravating infection.

To further verify the role of RNF5 in HSK, we used RNF5-siRNA to locally restrict the expression of RNF5 in the mouse cornea and then inoculated the mice with HSV-1 to establish an HSK epithelitis model. The subsequent analysis of the clinical scores and tear virus titers showed that RNF5 silencing effectively limited viral replication at the site of infection and alleviated HSK symptoms. In addition, PCR assays showed that the expression level of IFN-β was significantly increased after RNF5 silencing.

Studies have shown that HSV-1 infection will cause a significant increase in the secretion of IL-6 and TNF-α, which will aggravate the severity of HSK. IL-6 can stimulate corneal cells to produce macrophage inflammatory protein (MIP) and then promote the infiltration of neutrophils into the cornea (Fenton et al., 2002), and can also promote the production of VEGF by corneal cells and inflammatory cells to aggravate the formation of neovascularization. The use of the TNF-α antibody can effectively reduce the degree of HSK corneal opacity (Keadle et al., 2000). Western blotting analysis of mouse corneal tissue confirmed this theory. This shows that RNF5 silencing can effectively inhibit viral replication, control the infection, slow down the secretion of proinflammatory cytokines, and reduce inflammatory-cell infiltration in the corneal tissue in the later stages of the disease, thereby relieving corneal tissue damage caused by excessive autoimmune responses in the late stage of HSK.

## Conclusion

Our study shows that HSV-1 infection can induce elevated expression of RNF5. A large amount of RNF5 inhibits STING/IRF3 signaling and reduces IFN-β secretion through the ubiquitination and degradation of STING and IRF3, thereby promoting viral replication and aggravating inflammation. Restricting the expression of RNF5 is expected to increase the antiviral immune response of the host and alleviate HSK.

## Data availability statement

The original contributions presented in the study are included in the article/Supplementary Material, further inquiries can be directed to the corresponding author.

## Ethics statement

The animal study was reviewed and approved by the Ethics Committee of Shengjing Hospital, China Medical University (Ethics Number: 2021PS470K).

## Author contributions

ZL: conceptualization, formal analysis, investigation, methodology, supervision, visualization, writing – original draft, and writing – review and editing. LX: conceptualization, funding acquisition, methodology, supervision, writing – original draft, and writing – review and editing. Both authors contributed to the article and approved the submitted version.
