# Supplementary material for: E3 ligase RNF5 inhibits type I interferon response in herpes simplex virus keratitis through the STING/IRF3 signaling pathway
Source: Front Microbiol. 2022 Aug 2;13:944101. doi: 10.3389/fmicb.2022.944101 (PMC9382029; doi:10.3389/fmicb.2022.944101)
Supplement: Supplementary file 2 [file Presentation_1.PPTX]

## Slide 1
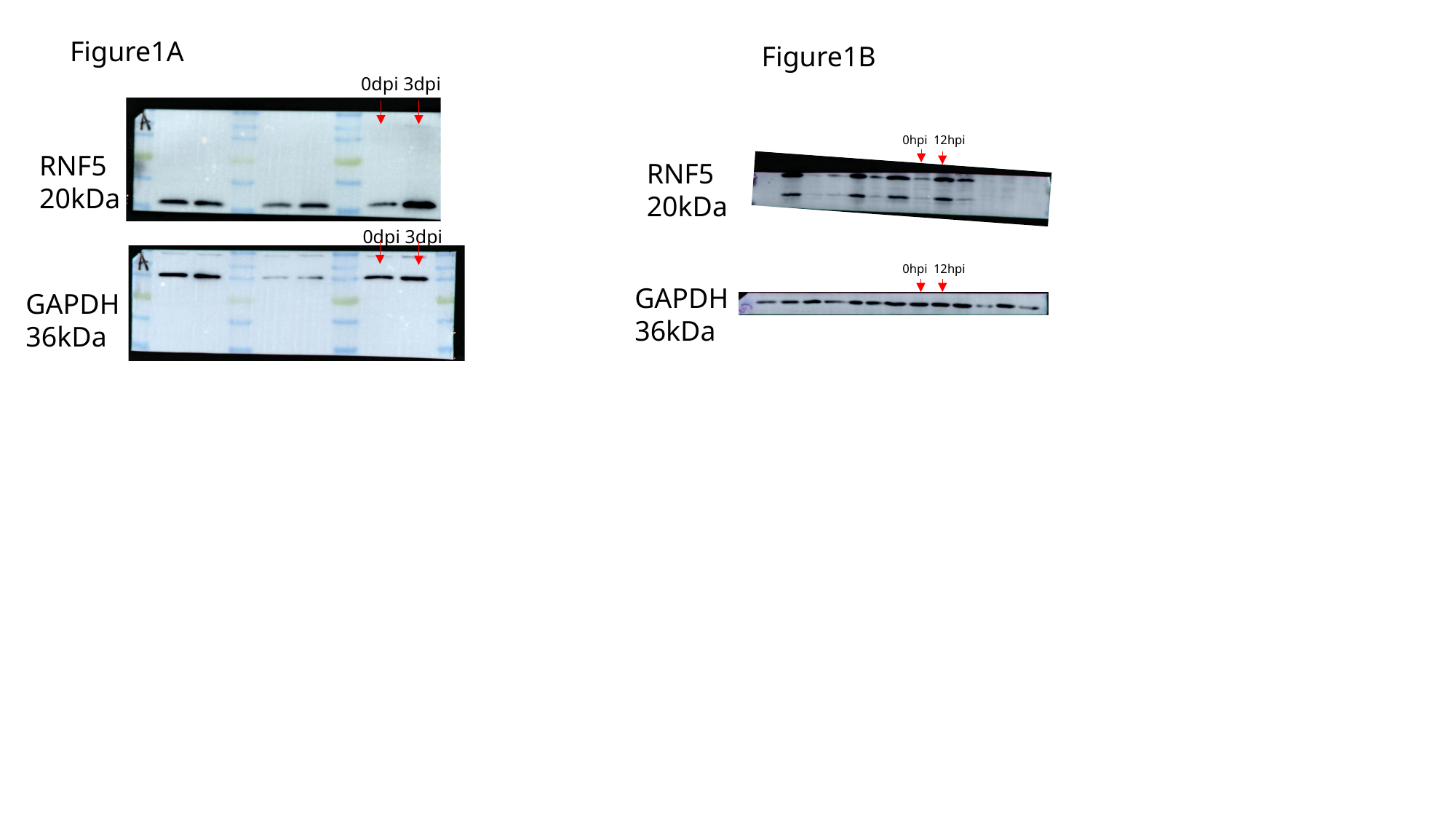

Figure1A
Figure1B
0dpi 3dpi
0hpi 12hpi
RNF5
20kDa
RNF5
20kDa
0dpi 3dpi
0hpi 12hpi
GAPDH
36kDa
GAPDH
36kDa

## Slide 2
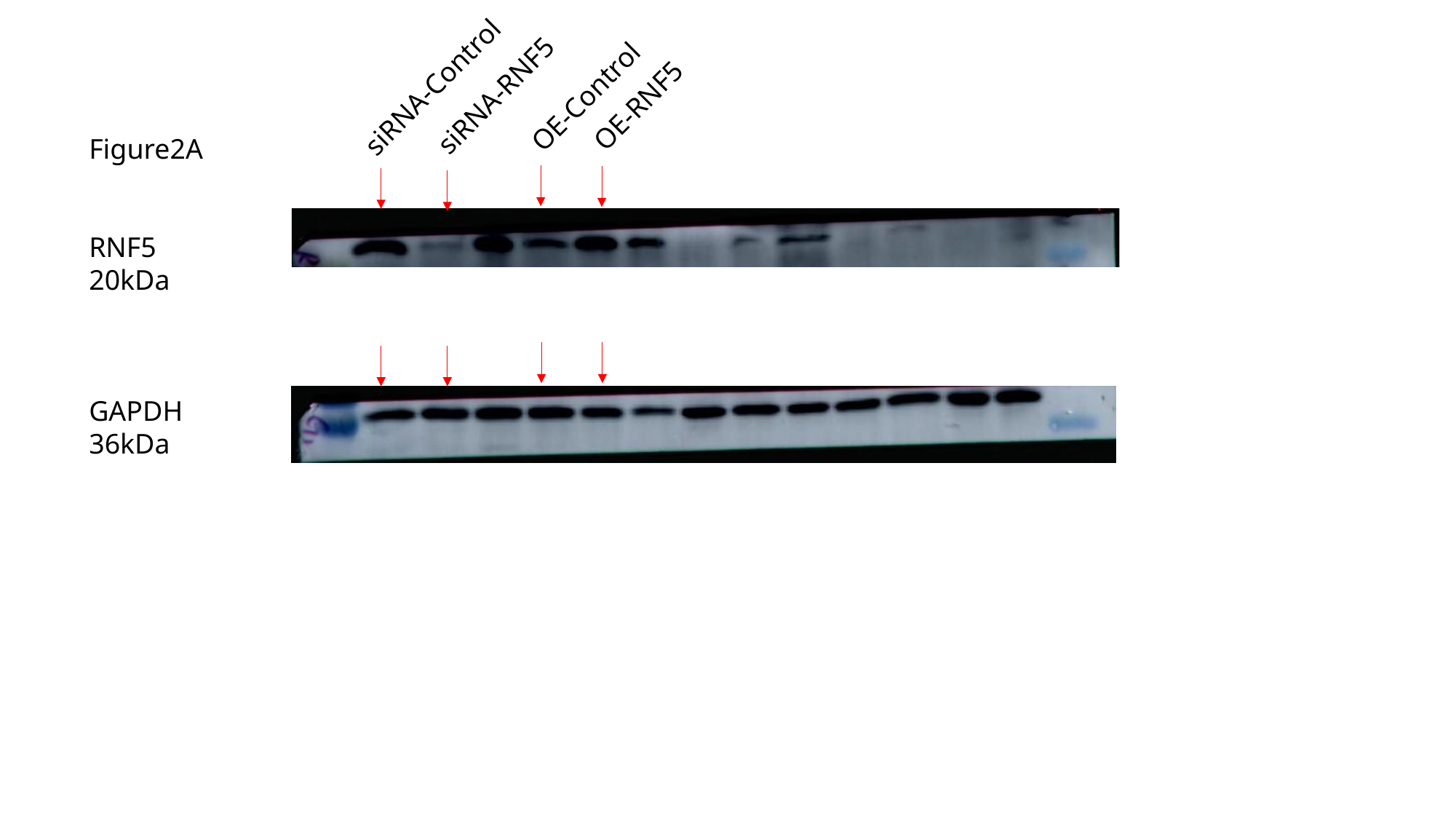

siRNA-Control
siRNA-RNF5
OE-Control
OE-RNF5
Figure2A
RNF5
20kDa
GAPDH
36kDa

## Slide 3
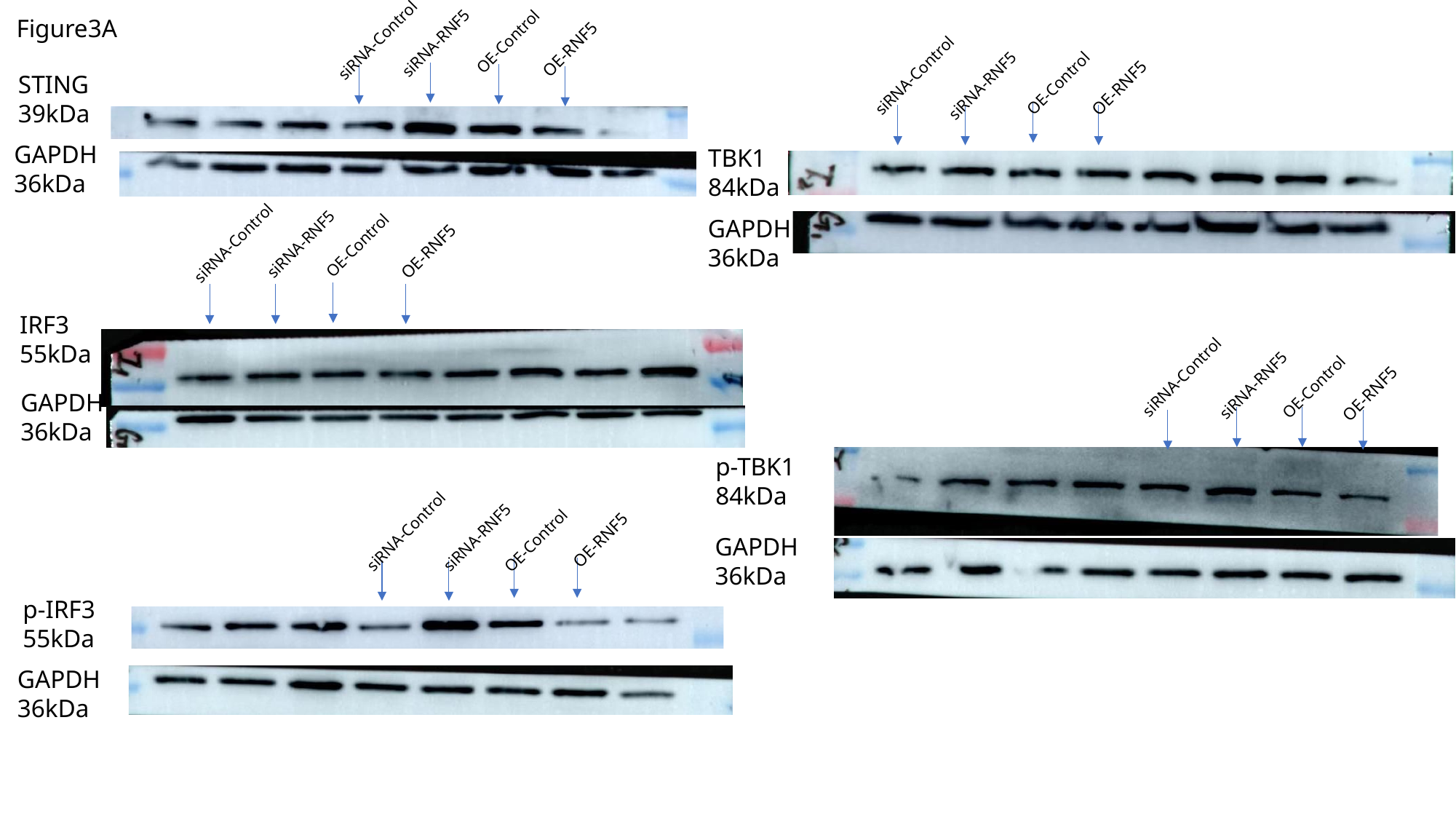

siRNA-RNF5
Figure3A
siRNA-Control
OE-Control
OE-RNF5
siRNA-RNF5
siRNA-Control
STING
39kDa
OE-Control
OE-RNF5
GAPDH
36kDa
TBK1
84kDa
siRNA-RNF5
GAPDH
36kDa
siRNA-Control
OE-Control
OE-RNF5
IRF3
55kDa
siRNA-RNF5
siRNA-Control
OE-Control
OE-RNF5
GAPDH
36kDa
p-TBK1
84kDa
siRNA-RNF5
siRNA-Control
OE-RNF5
OE-Control
GAPDH
36kDa
p-IRF3
55kDa
GAPDH
36kDa

## Slide 4
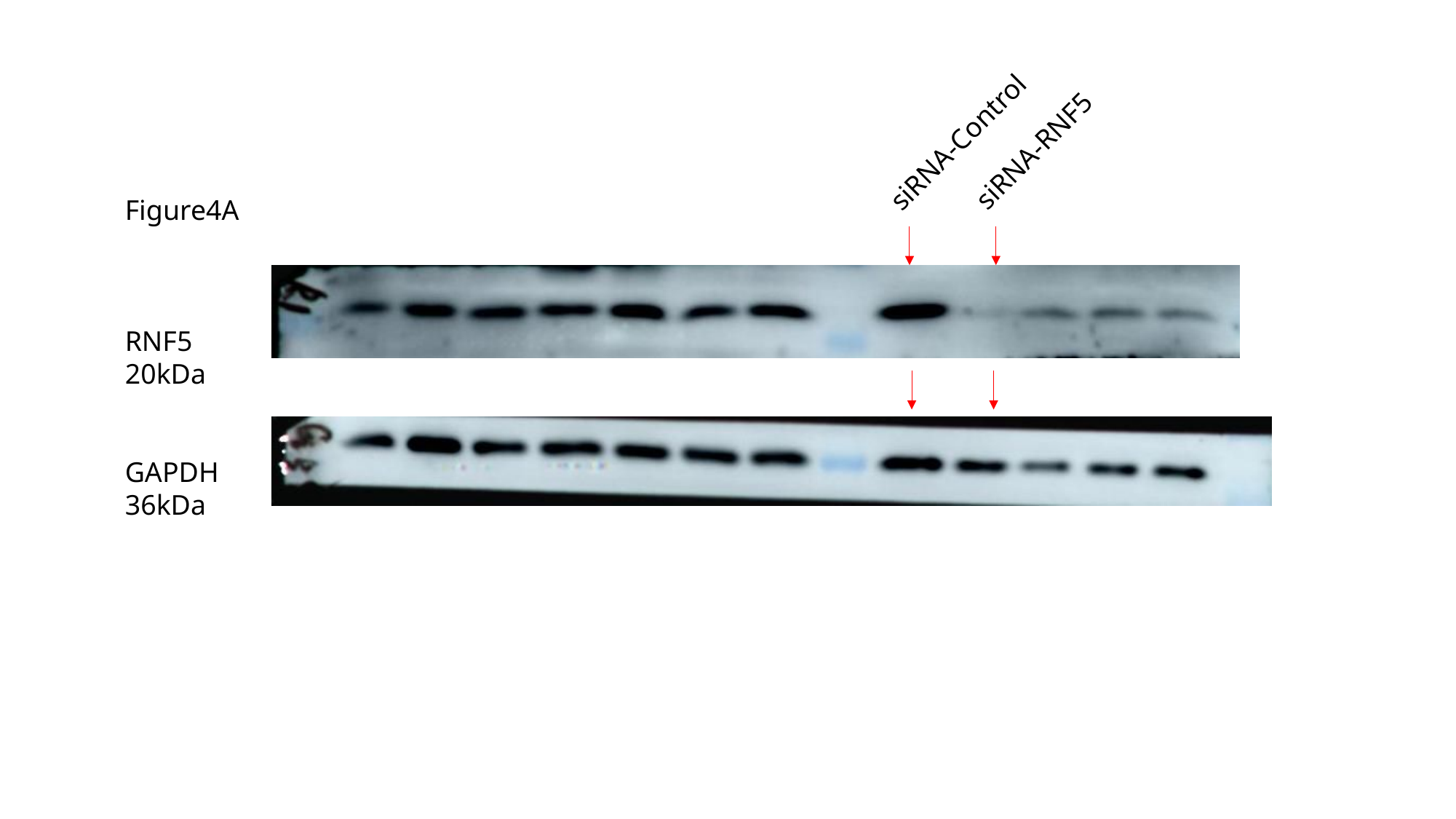

siRNA-Control
siRNA-RNF5
Figure4A
RNF5
20kDa
GAPDH
36kDa

## Slide 5
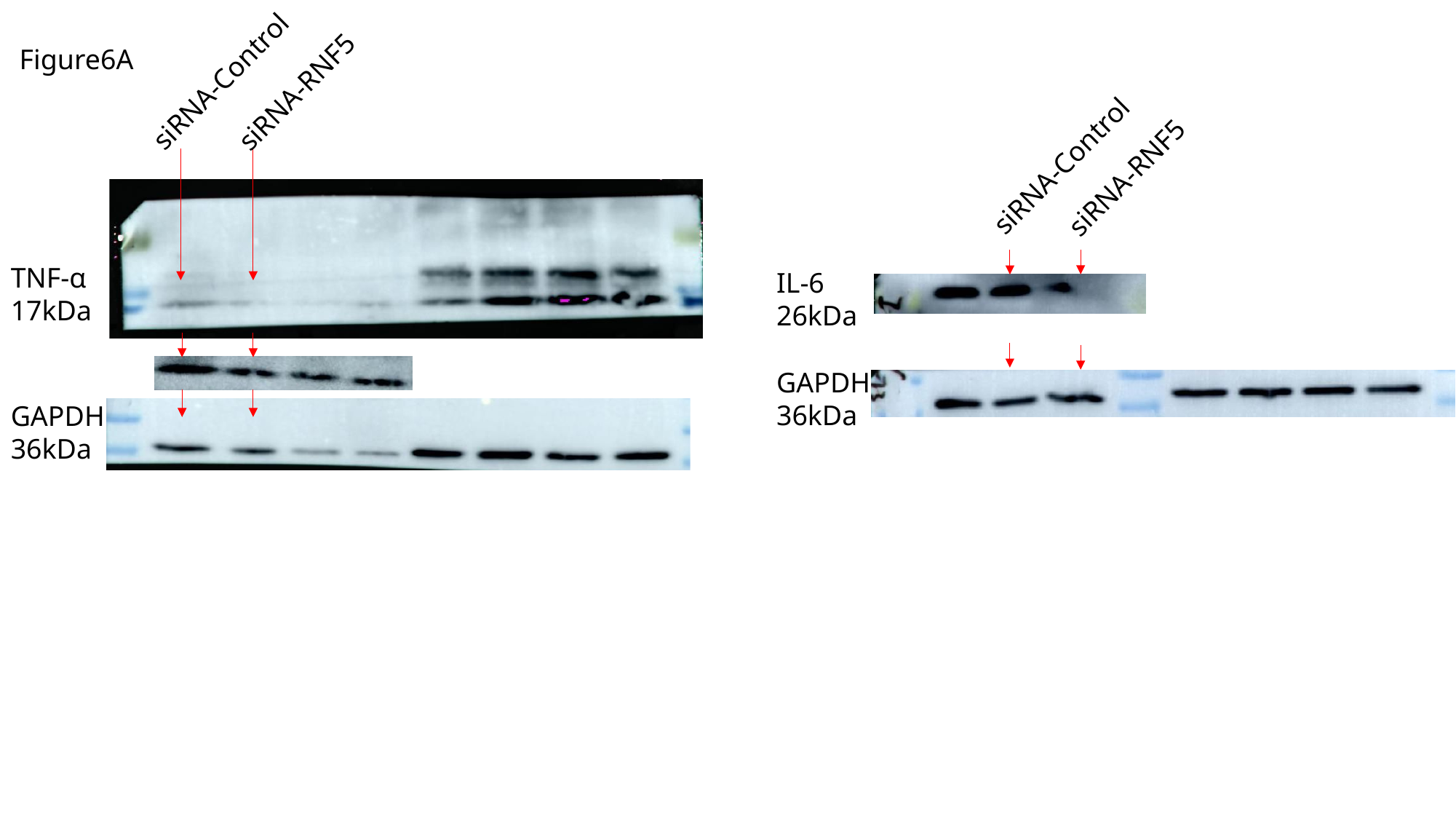

Figure6A
siRNA-Control
siRNA-RNF5
siRNA-Control
siRNA-RNF5
TNF-α
17kDa
IL-6
26kDa
GAPDH
36kDa
GAPDH
36kDa
