# Supplementary figures and images for: E3 ligase RNF5 inhibits type I interferon response in herpes simplex virus keratitis through the STING/IRF3 signaling pathway
Source: Front Microbiol. 2022 Aug 2;13:944101. doi: 10.3389/fmicb.2022.944101 (PMC9382029; doi:10.3389/fmicb.2022.944101)

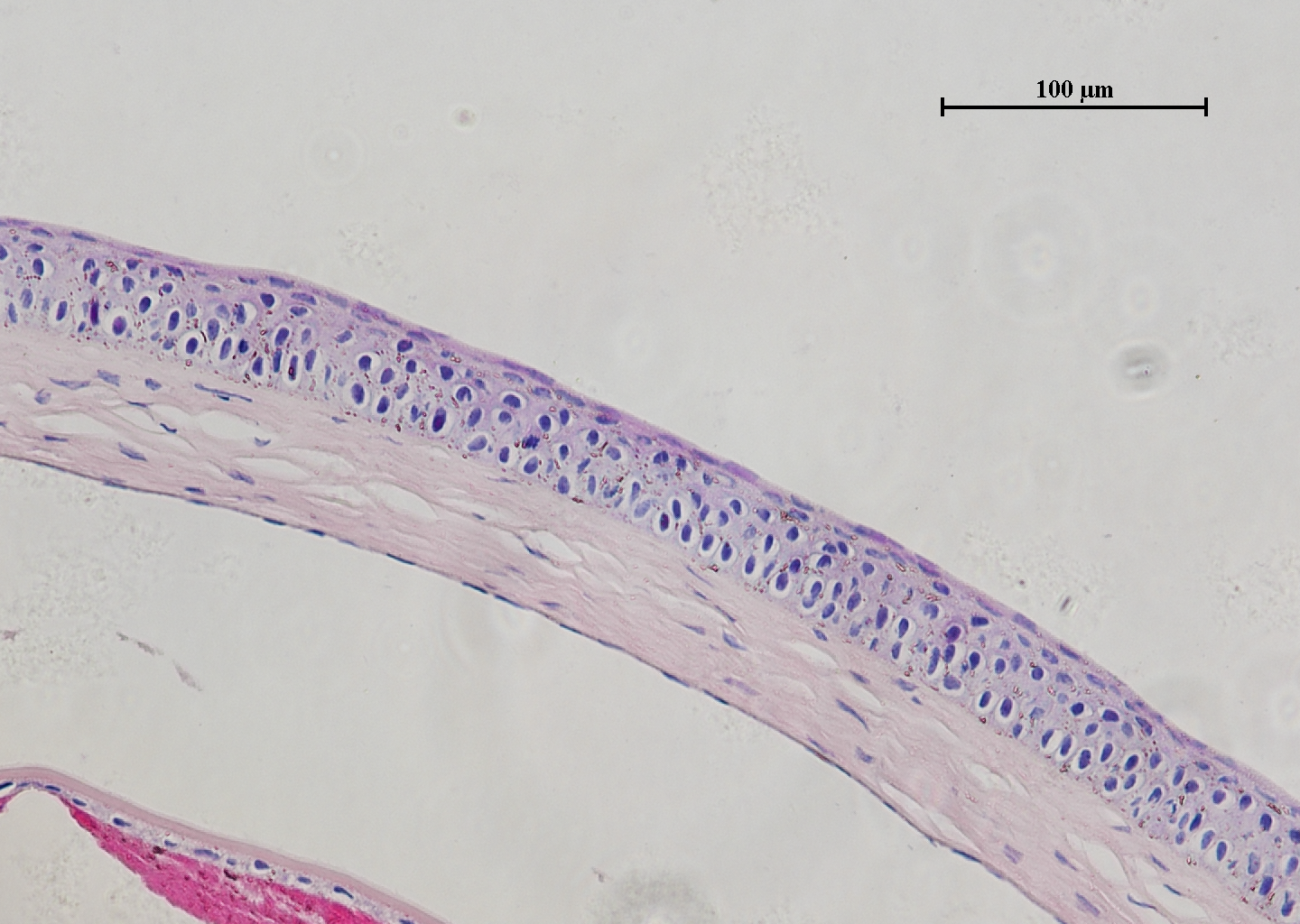

Supplement: Supplementary file 3 [file Data_Sheet_1.ZIP › Figure5/0dpi 200X HE.tif]

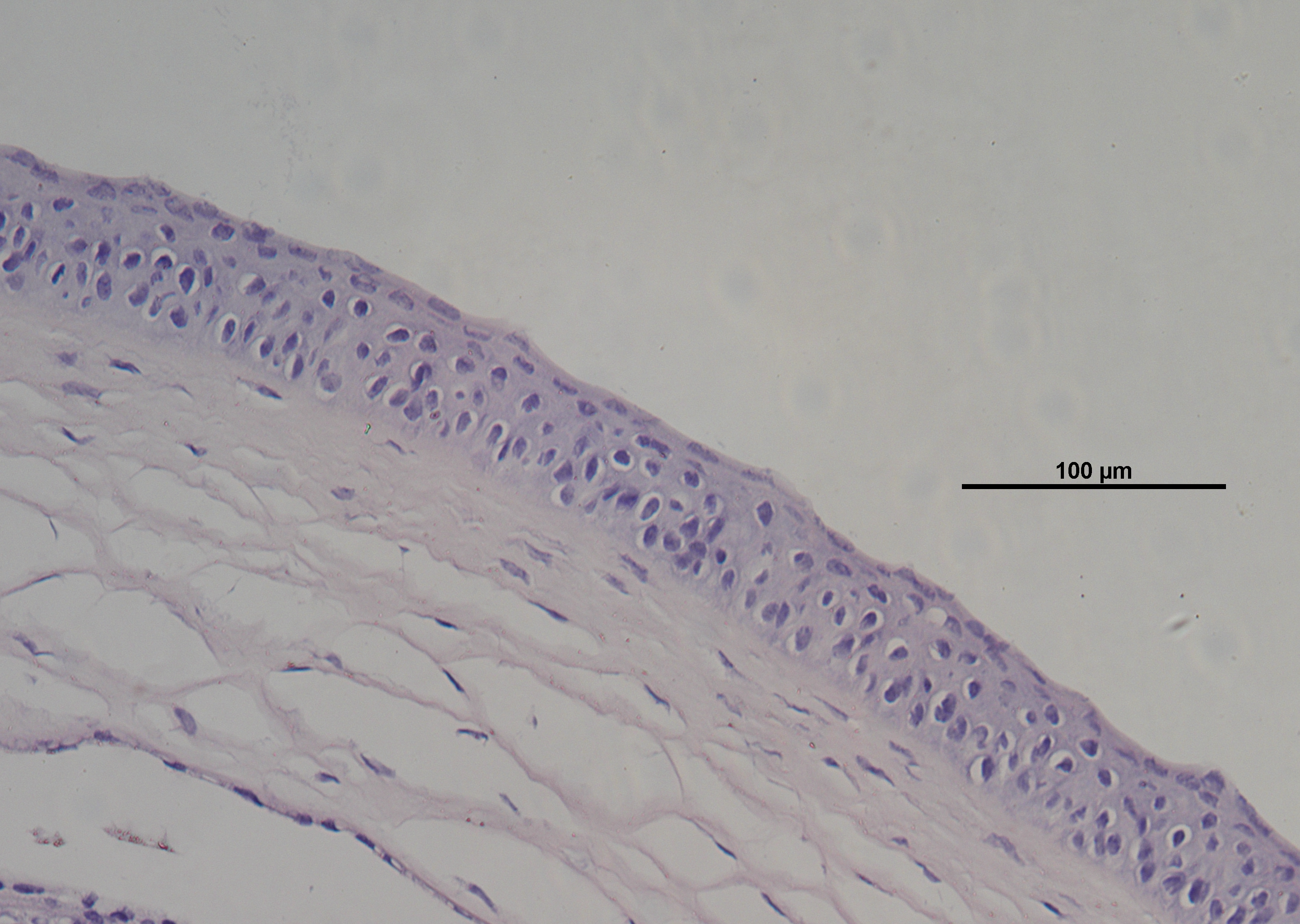

Supplement: Supplementary file 3 [file Data_Sheet_1.ZIP › Figure5/3dpi siRNA-RNF5 200X HE.tif]

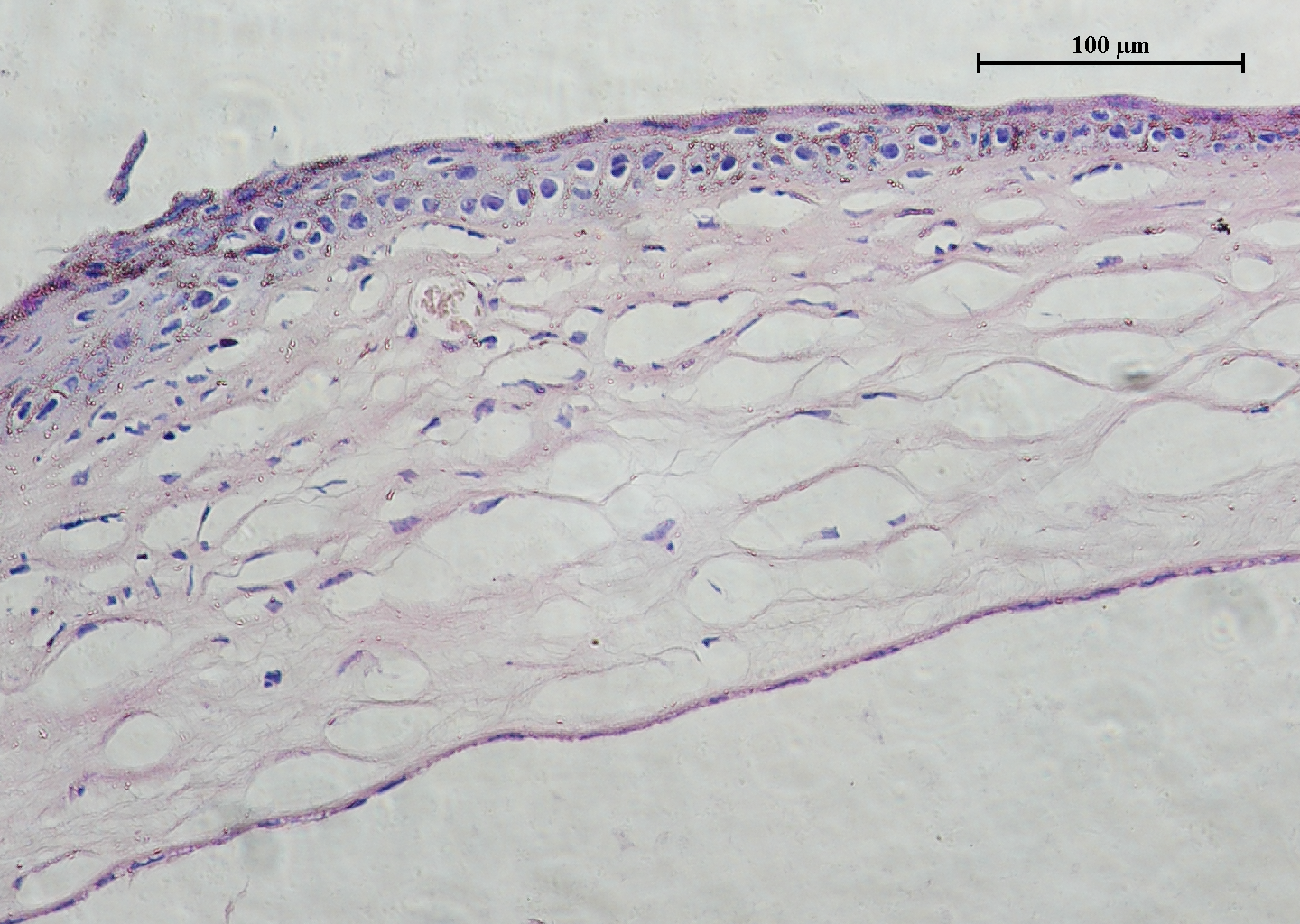

Supplement: Supplementary file 3 [file Data_Sheet_1.ZIP › Figure5/3dpi siRNA-control 200X HE.tif]

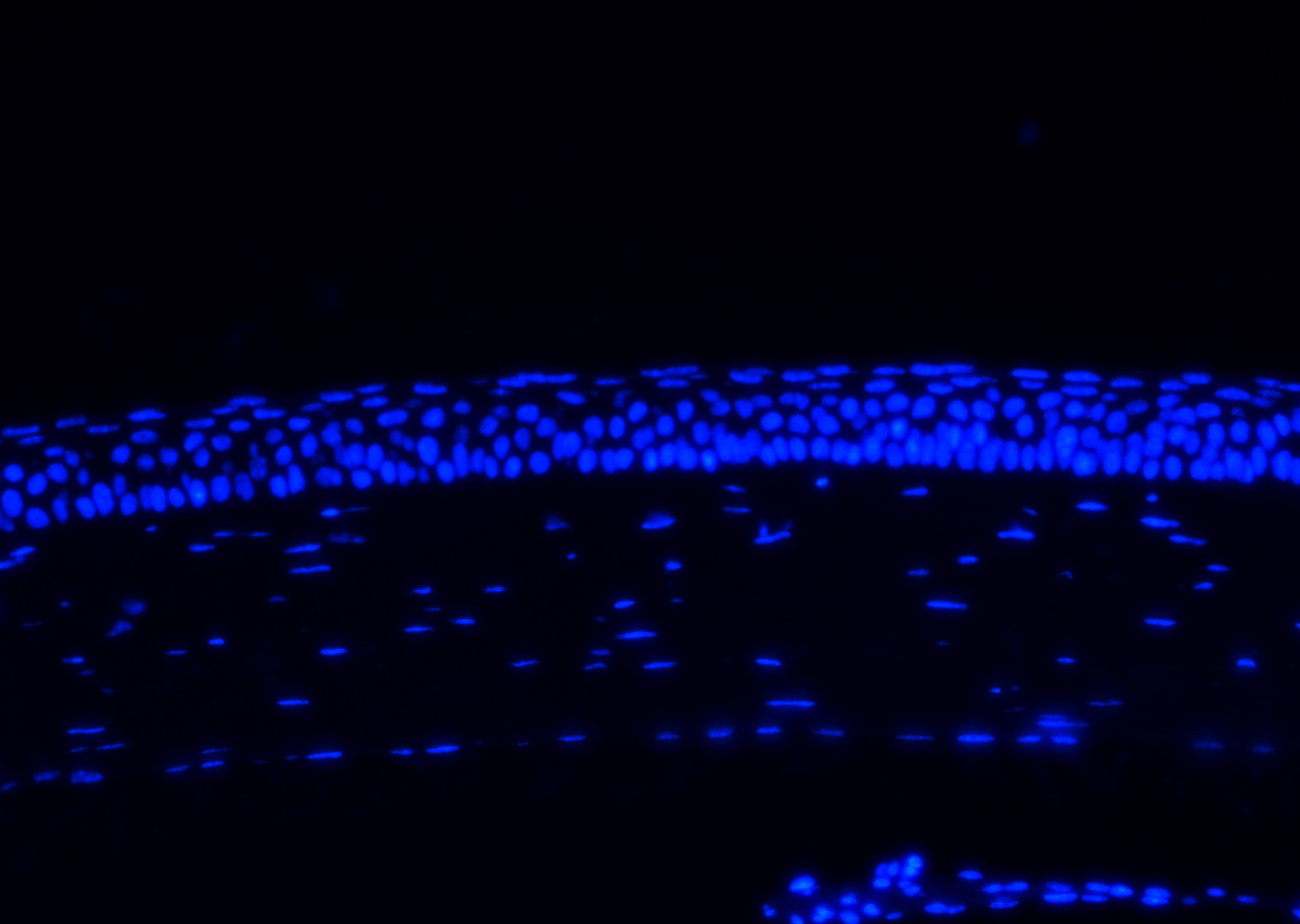

Supplement: Supplementary file 4 [file Data_Sheet_2.ZIP › 0dpi 200X DAPI.tif]

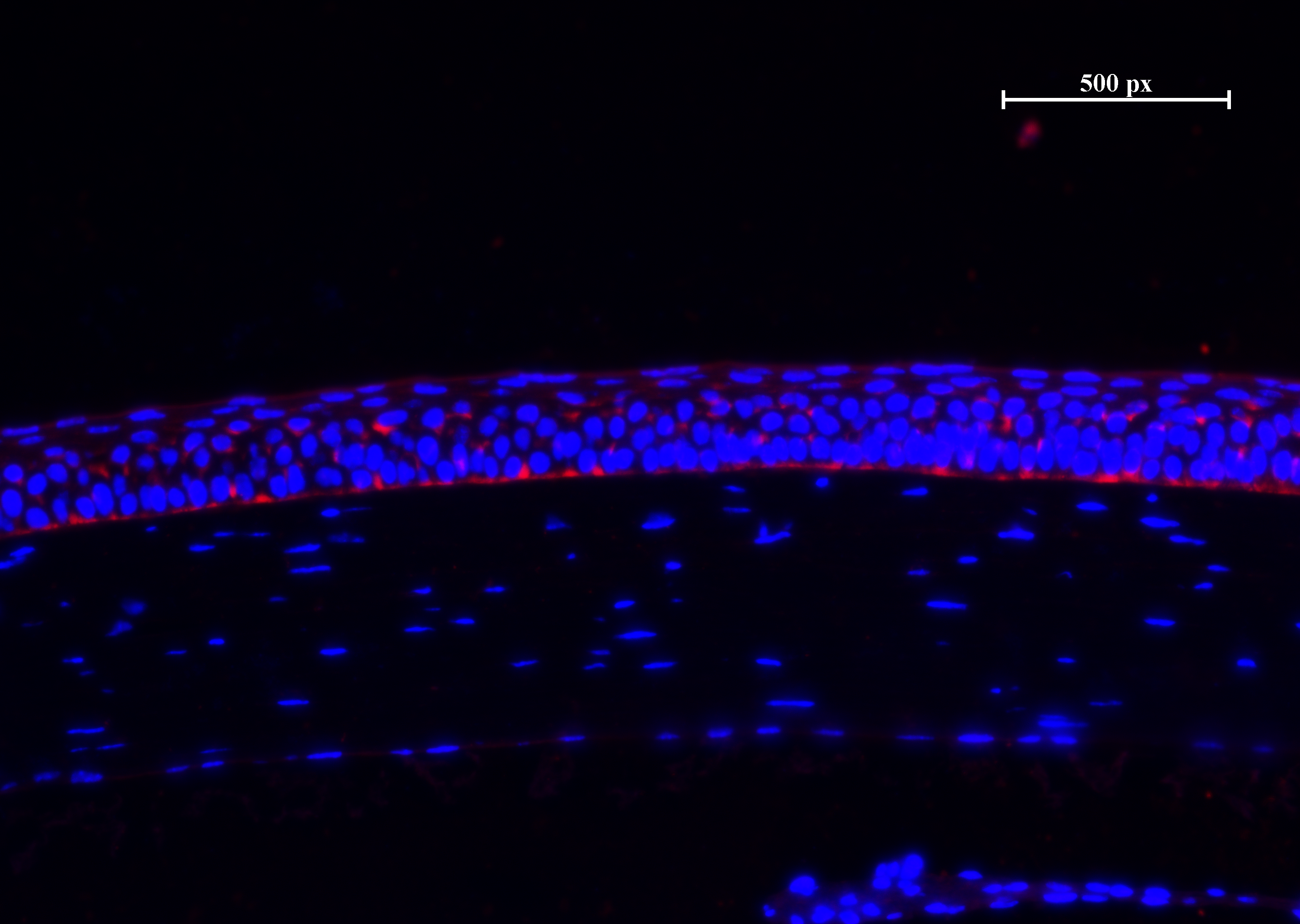

Supplement: Supplementary file 4 [file Data_Sheet_2.ZIP › 0dpi 200X Merged.tif]

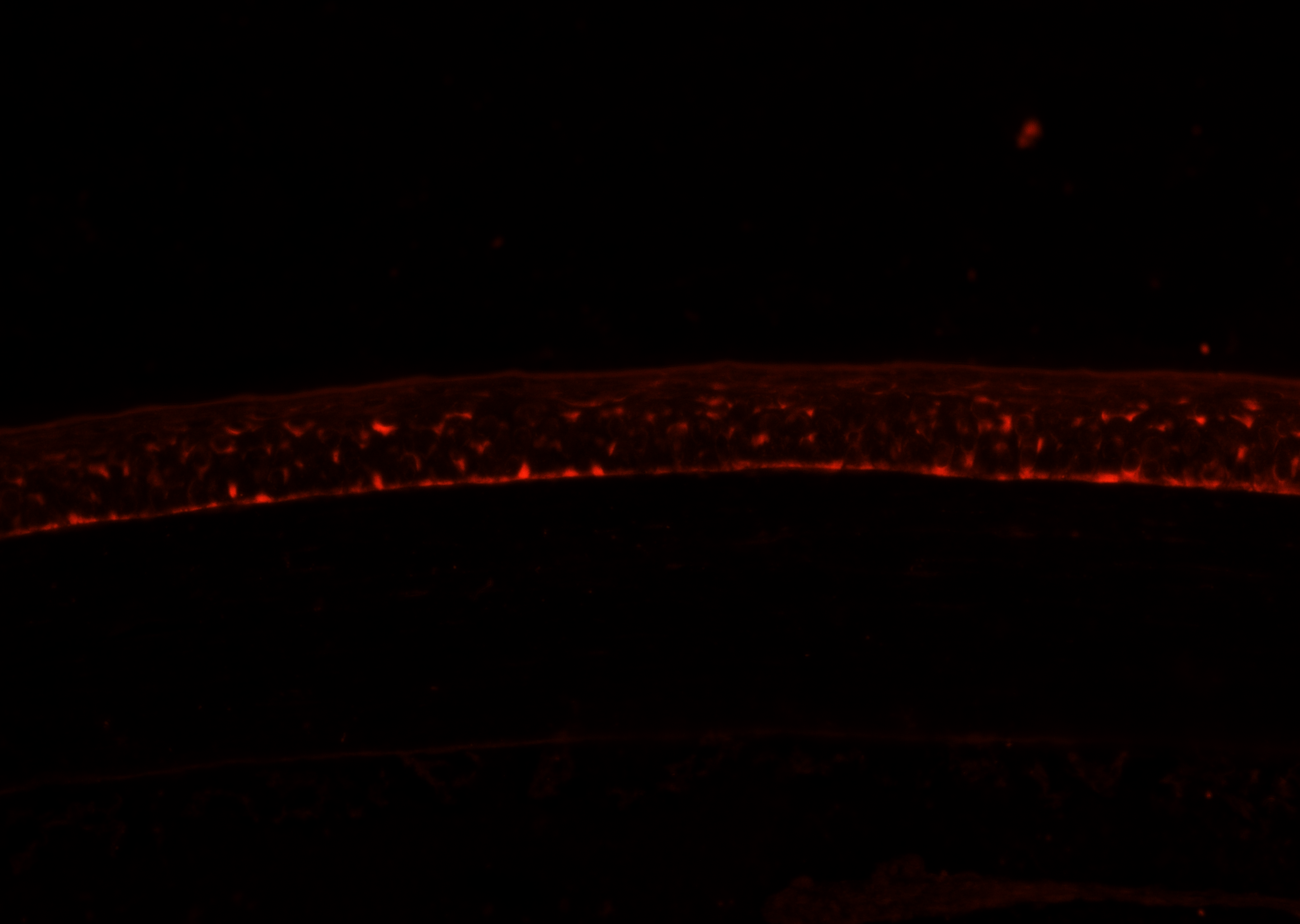

Supplement: Supplementary file 4 [file Data_Sheet_2.ZIP › 0dpi 200X SABC-CY3.tif]

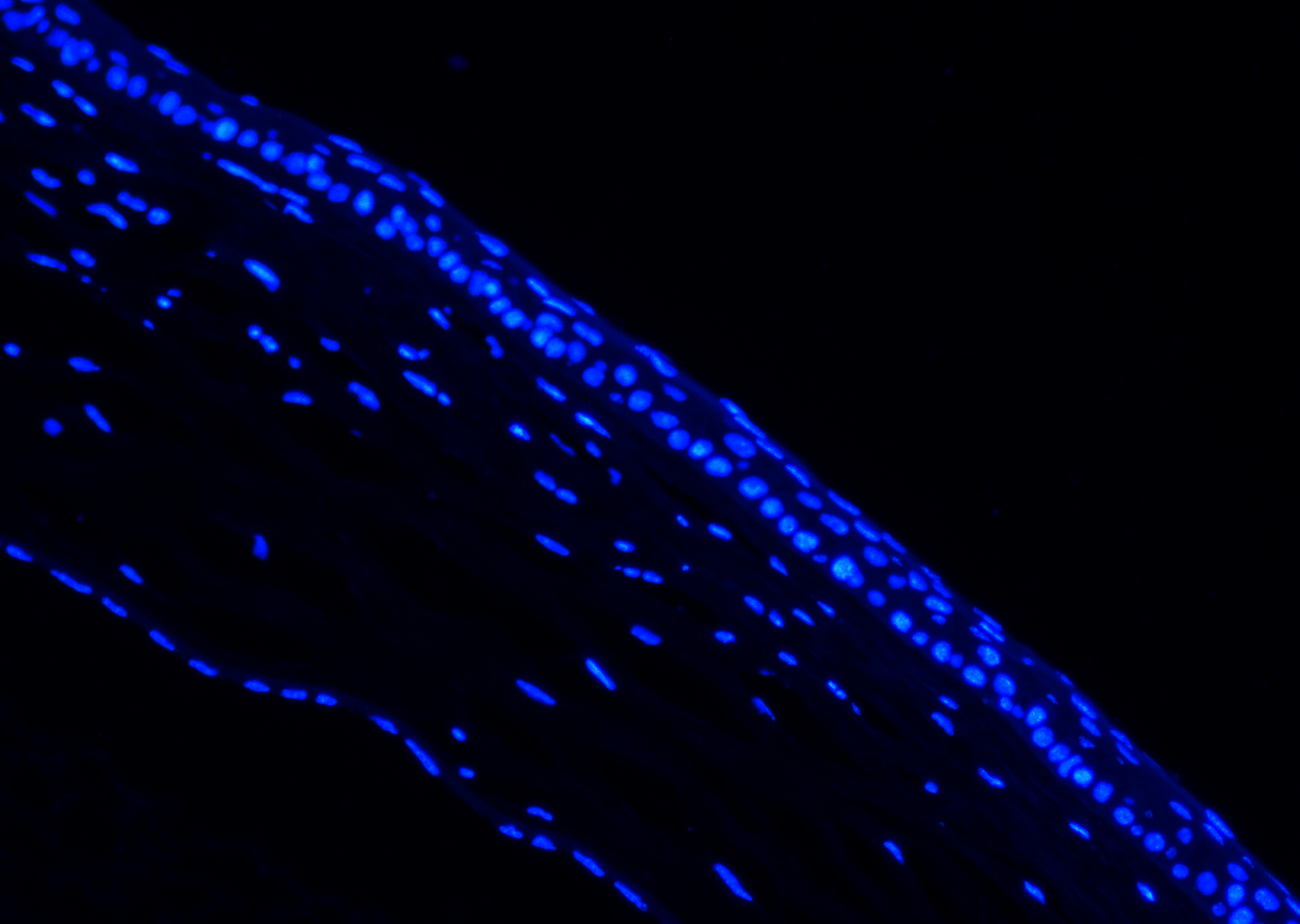

Supplement: Supplementary file 5 [file Data_Sheet_3.ZIP › 3dpi 200X DAPI.tif]

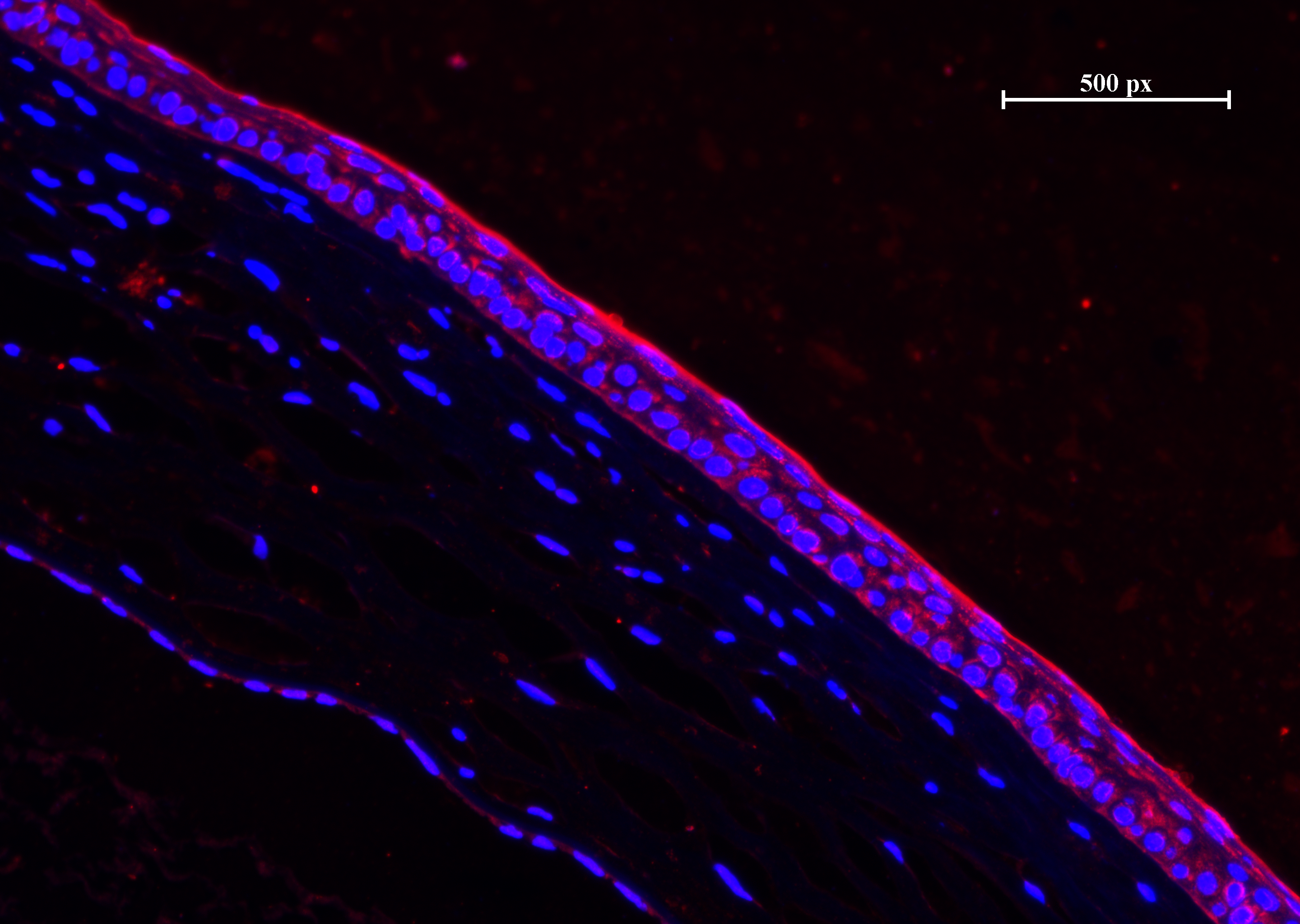

Supplement: Supplementary file 5 [file Data_Sheet_3.ZIP › 3dpi 200X Merged.tif]

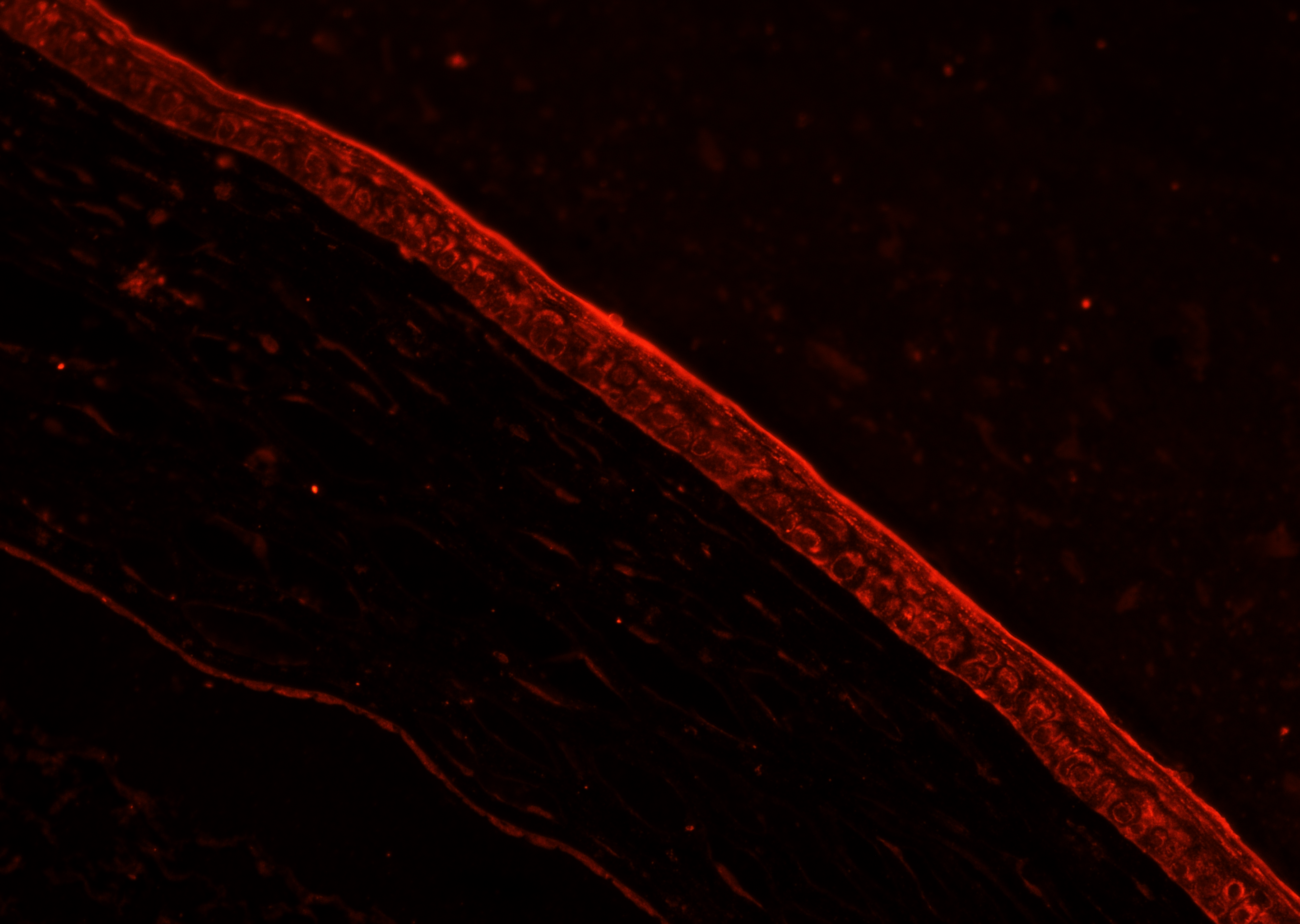

Supplement: Supplementary file 5 [file Data_Sheet_3.ZIP › 3dpi 200X SABC-CY3.tif]

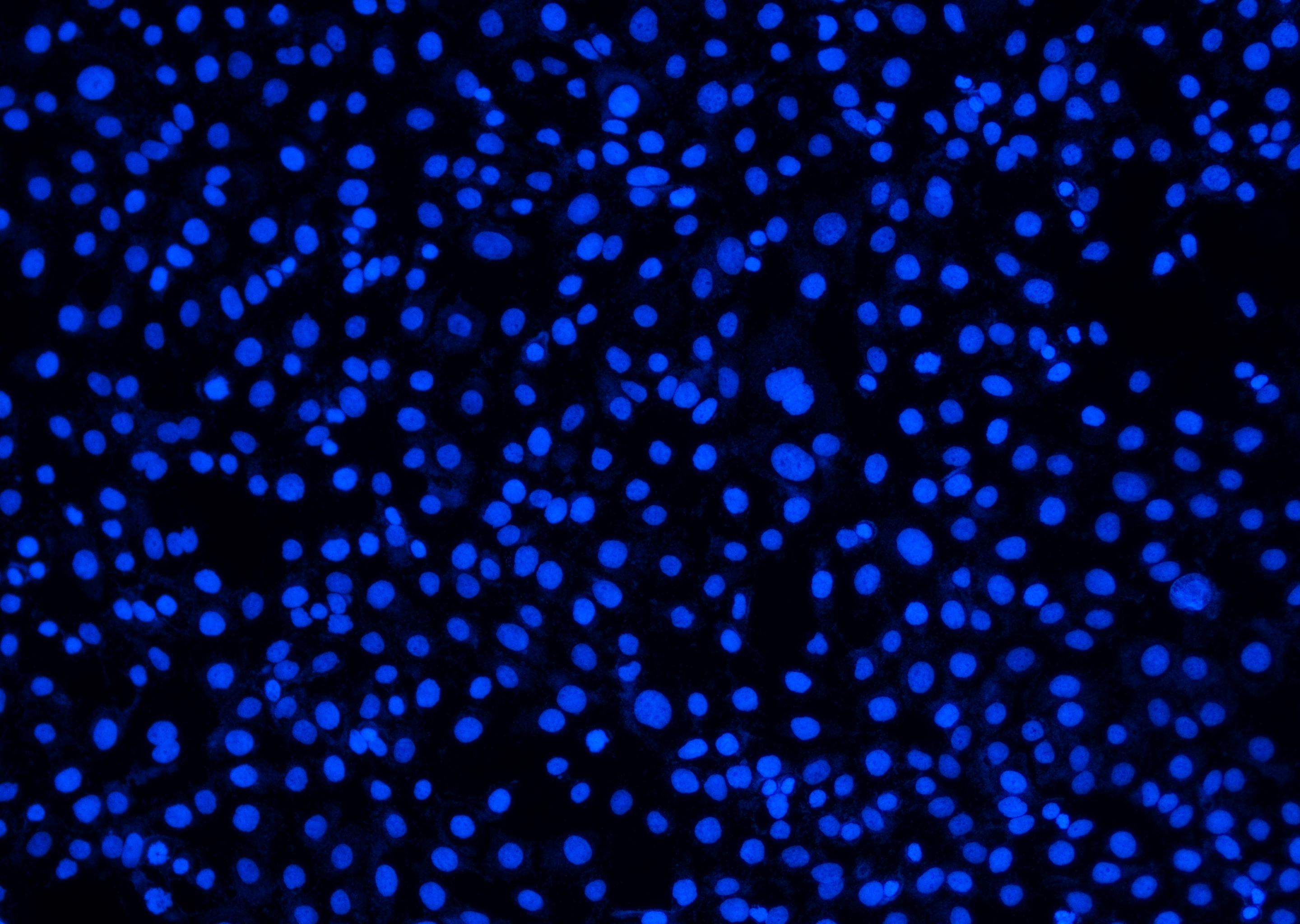

Supplement: Supplementary file 6 [file Data_Sheet_4.ZIP › 0hpi 100X DAPI.tif]

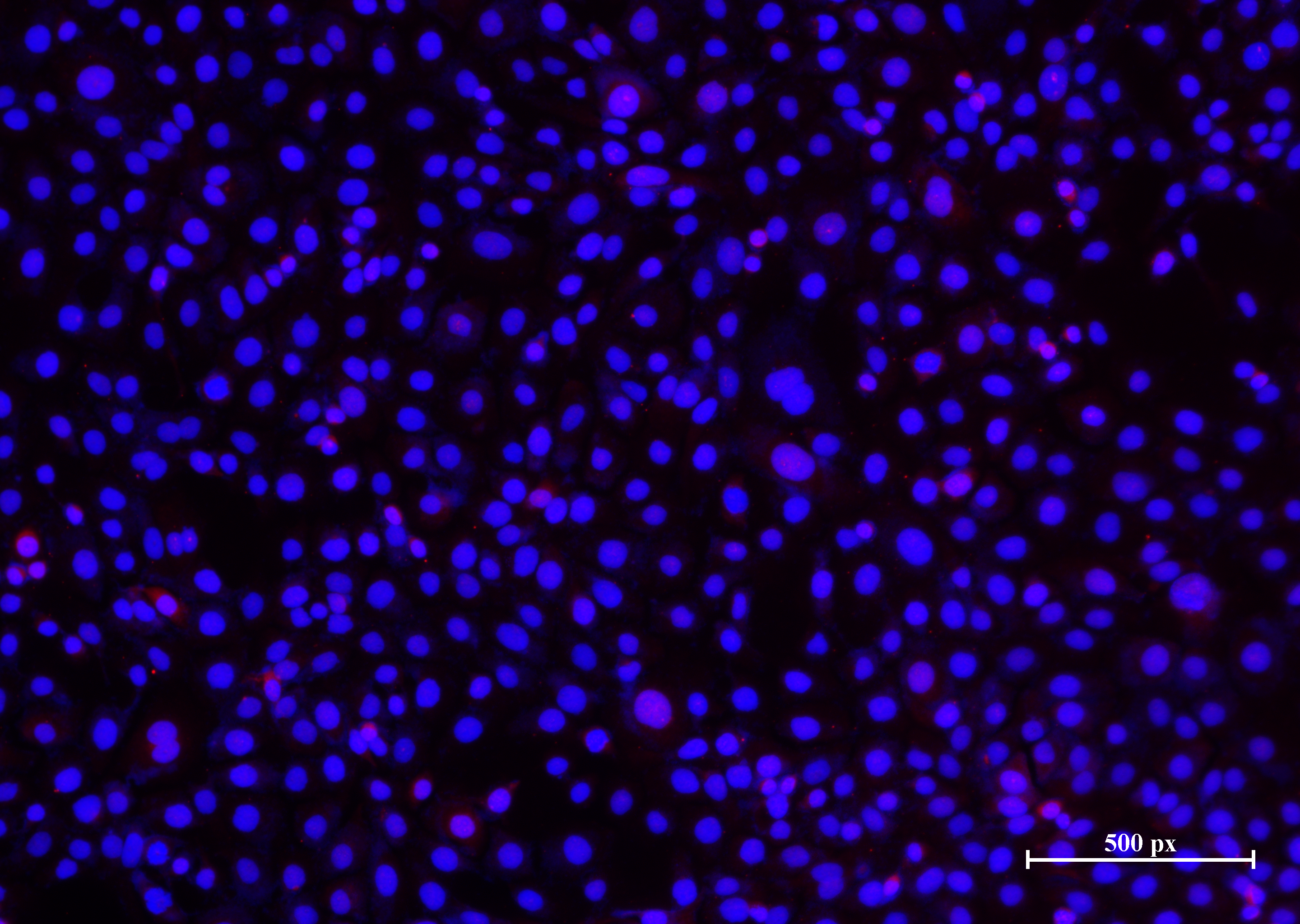

Supplement: Supplementary file 6 [file Data_Sheet_4.ZIP › 0hpi 100X Merged.tif]

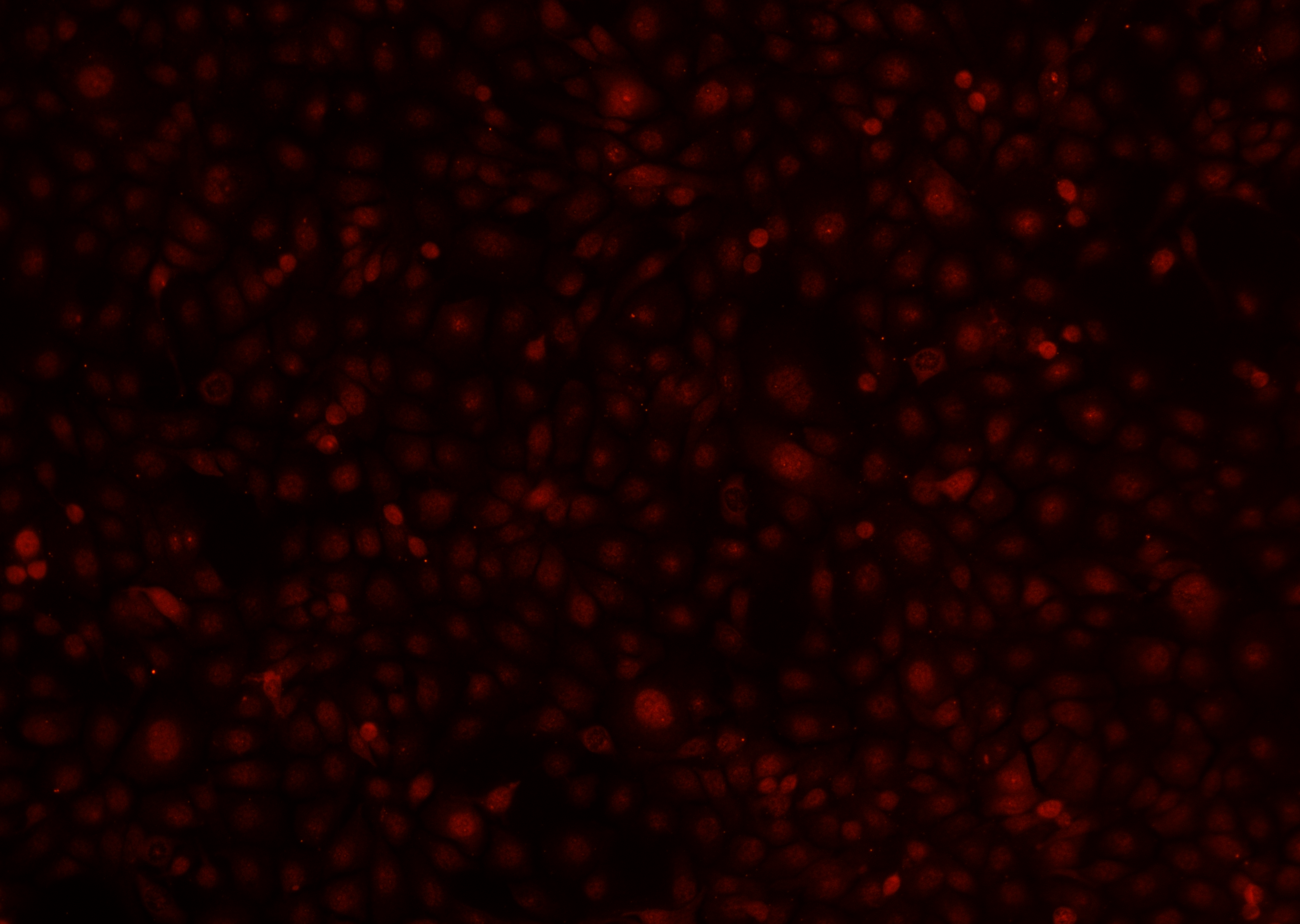

Supplement: Supplementary file 6 [file Data_Sheet_4.ZIP › 0hpi 100X SABC-CY3.tif]

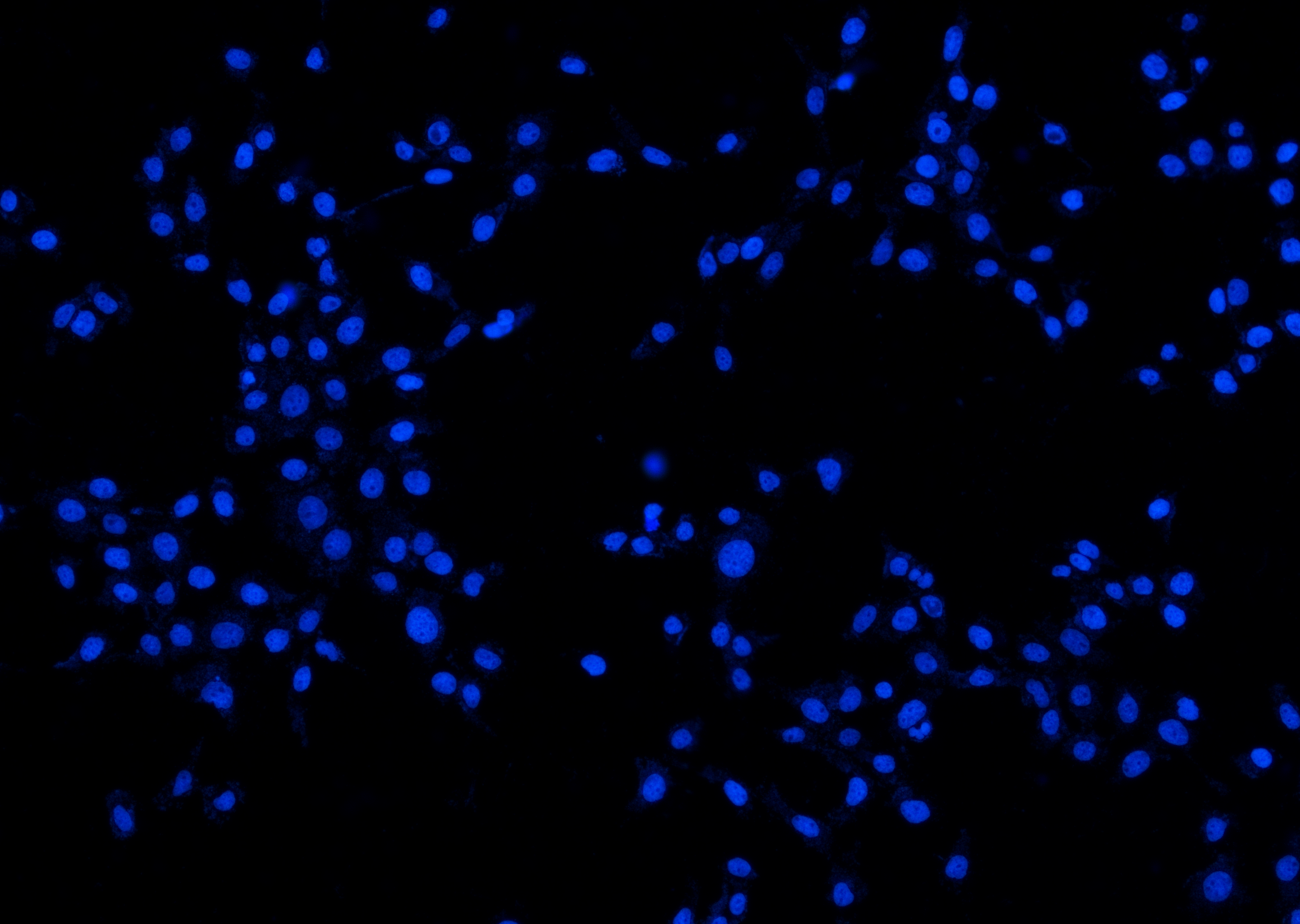

Supplement: Supplementary file 7 [file Data_Sheet_5.ZIP › 12hpi 100X DAPI.tif]

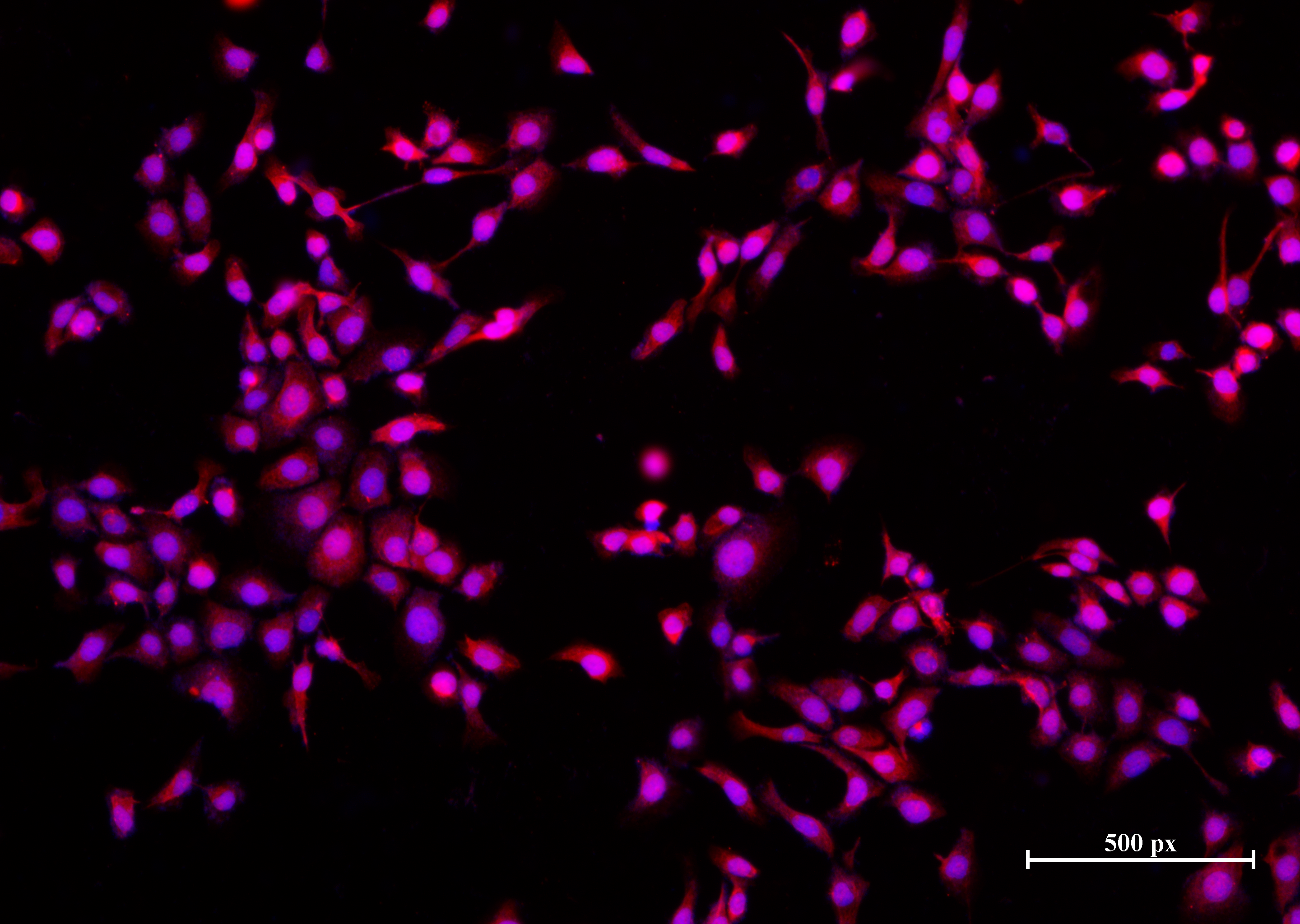

Supplement: Supplementary file 7 [file Data_Sheet_5.ZIP › 12hpi 100X Merged.tif]

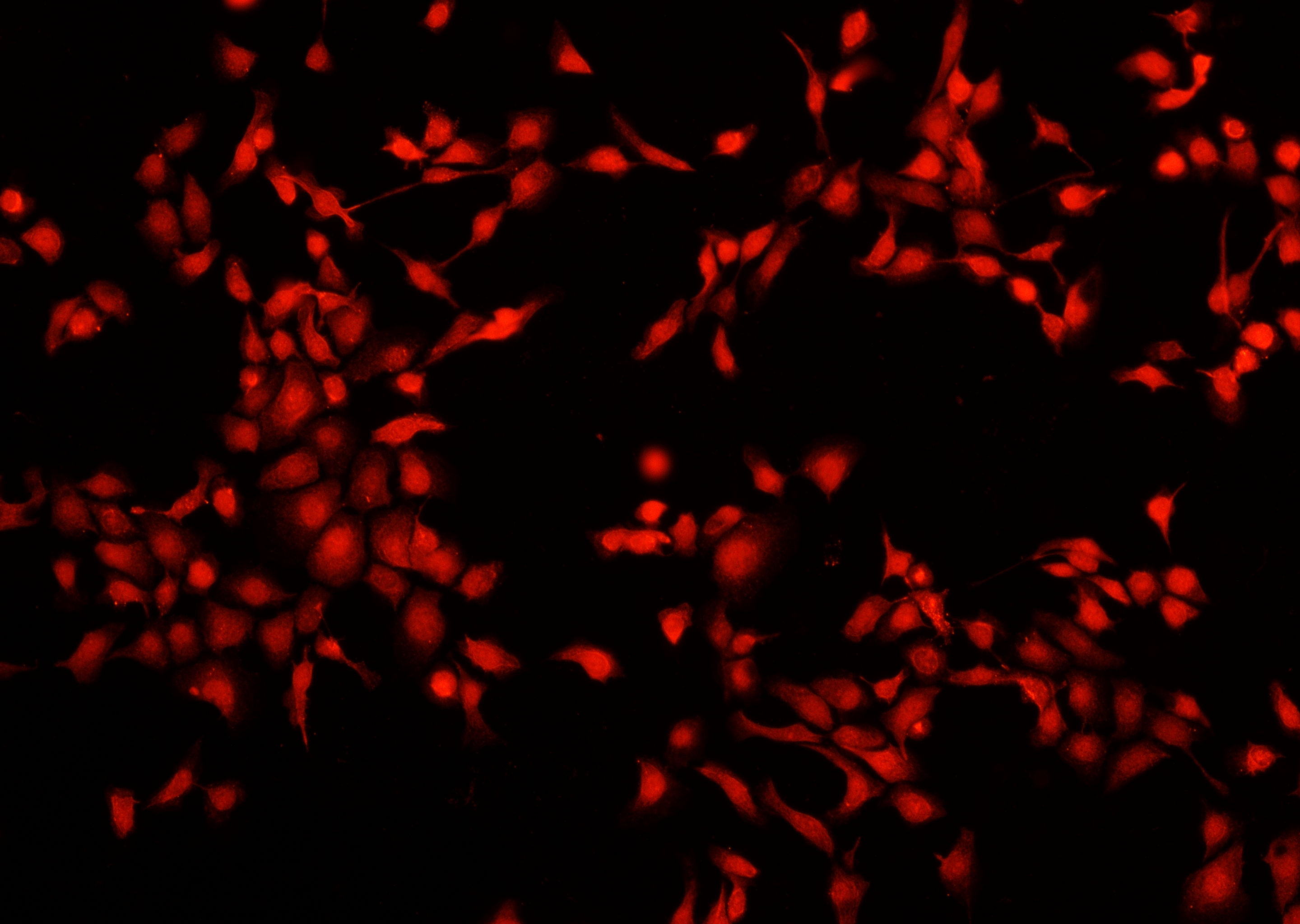

Supplement: Supplementary file 7 [file Data_Sheet_5.ZIP › 12hpi 100X SABC-CY3.tif]

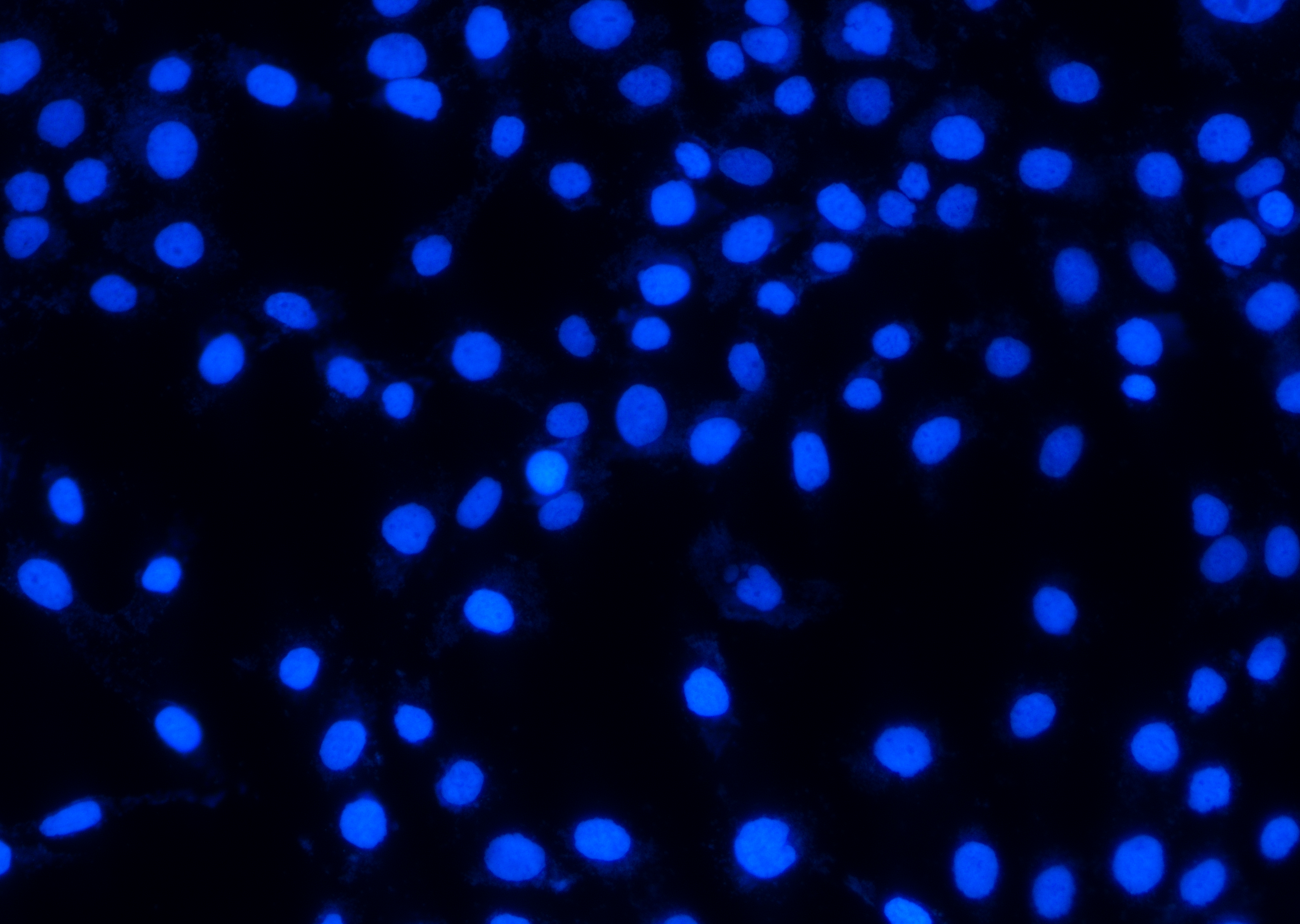

Supplement: Supplementary file 8 [file Data_Sheet_6.ZIP › 0hpi 200X DAPI.tif]

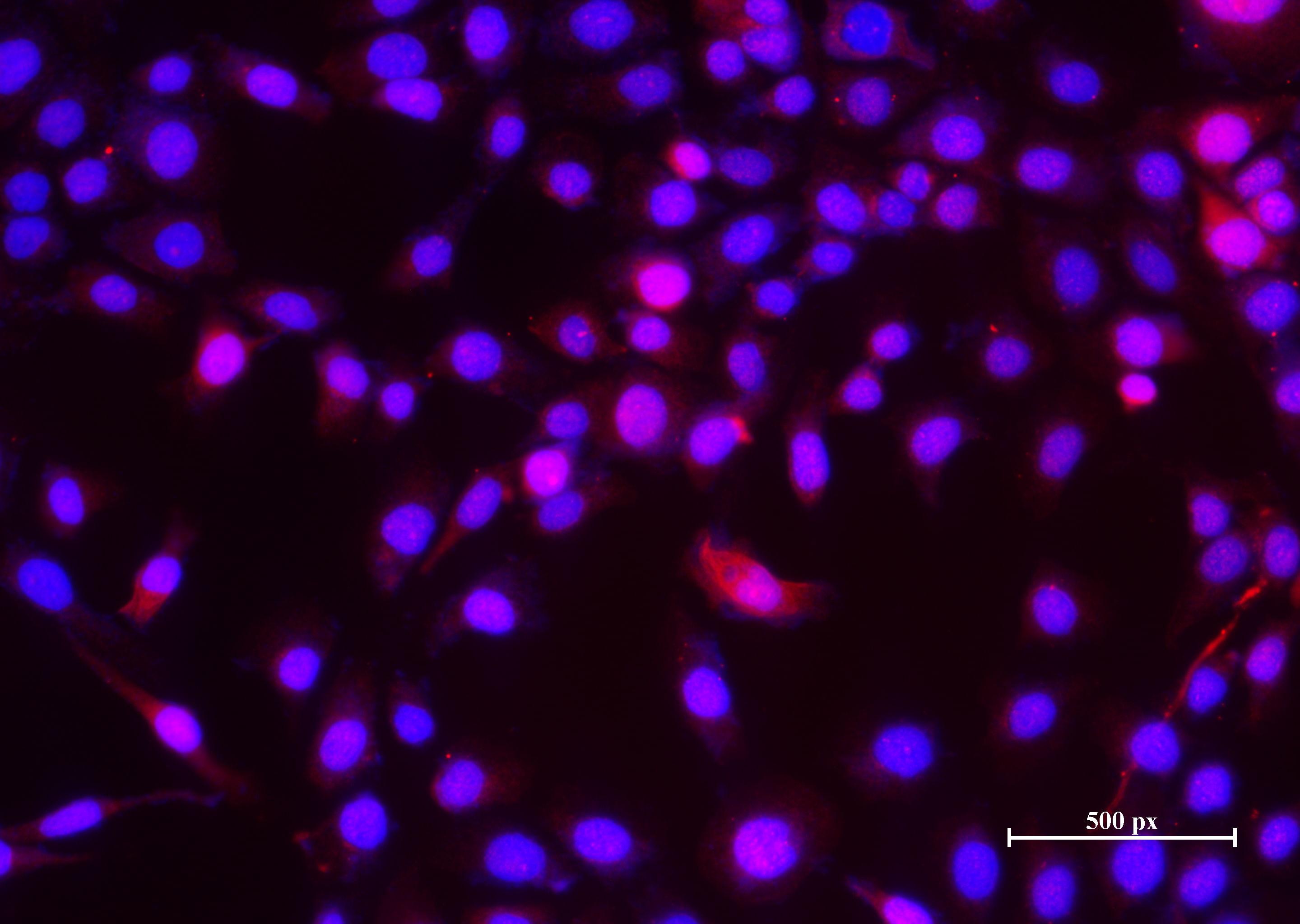

Supplement: Supplementary file 8 [file Data_Sheet_6.ZIP › 0hpi 200X Merged.tif]

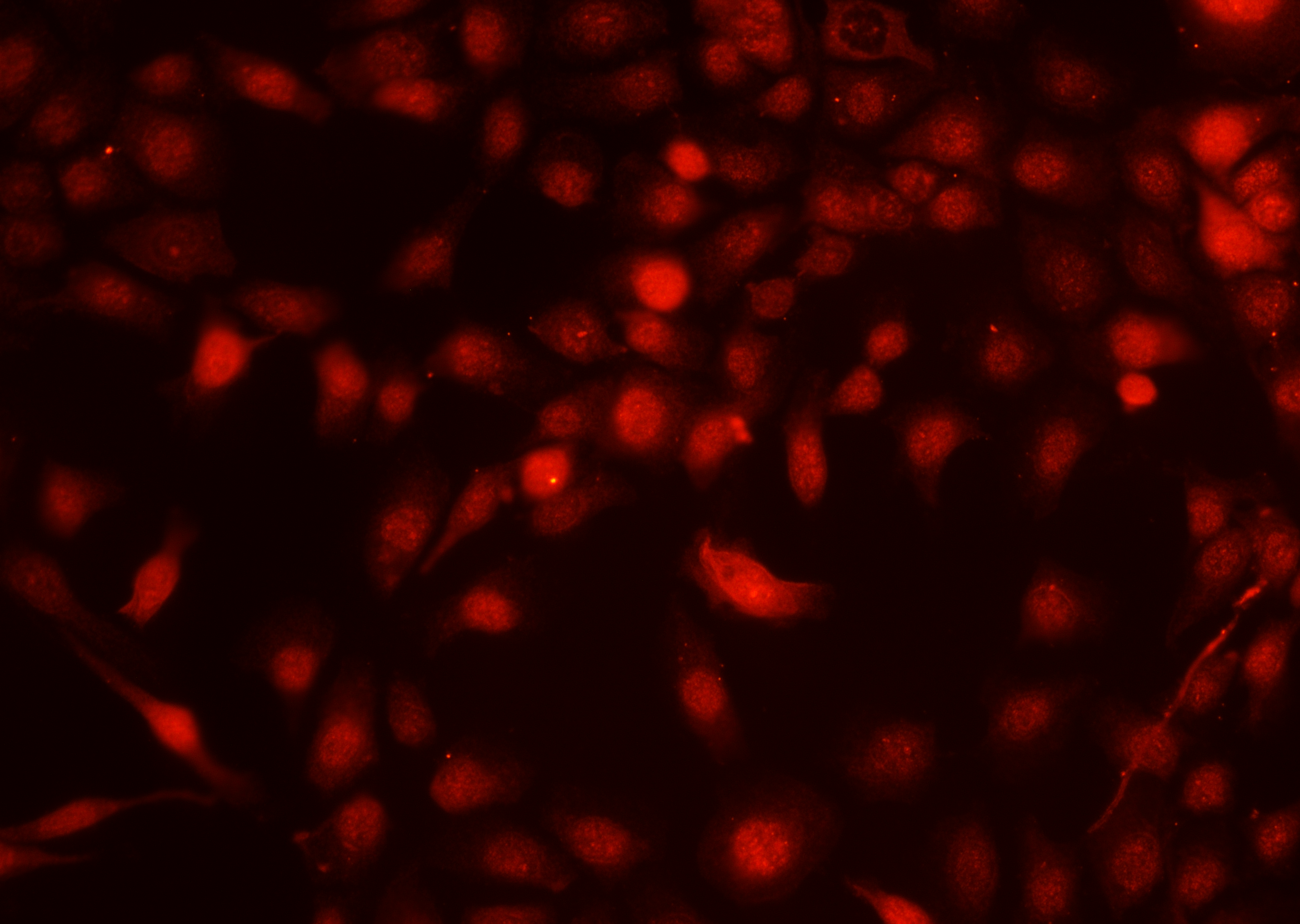

Supplement: Supplementary file 8 [file Data_Sheet_6.ZIP › 0hpi 200X SABC-CY3.tif]

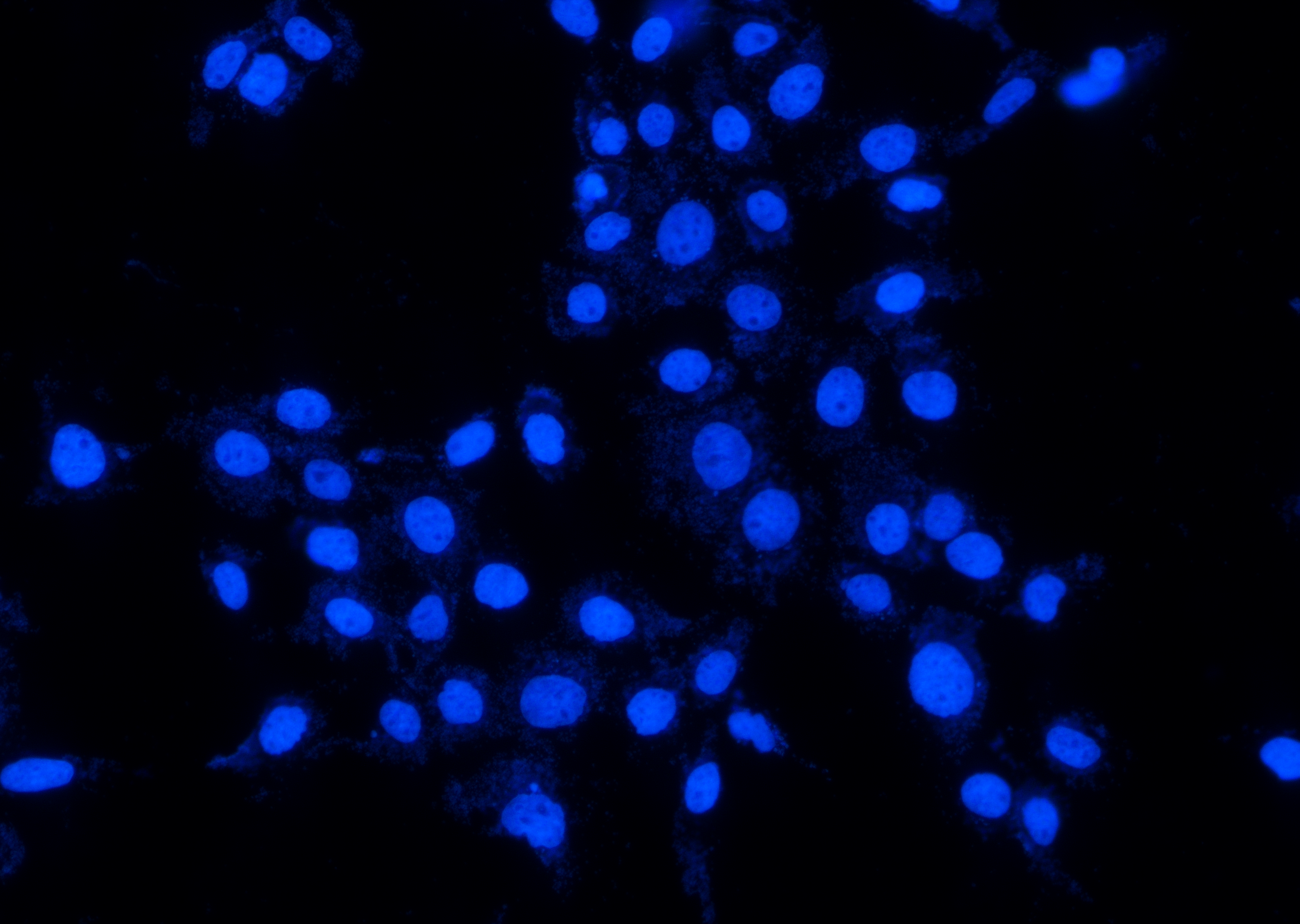

Supplement: Supplementary file 9 [file Data_Sheet_7.ZIP › 12hpi 200X DAPI.tif]

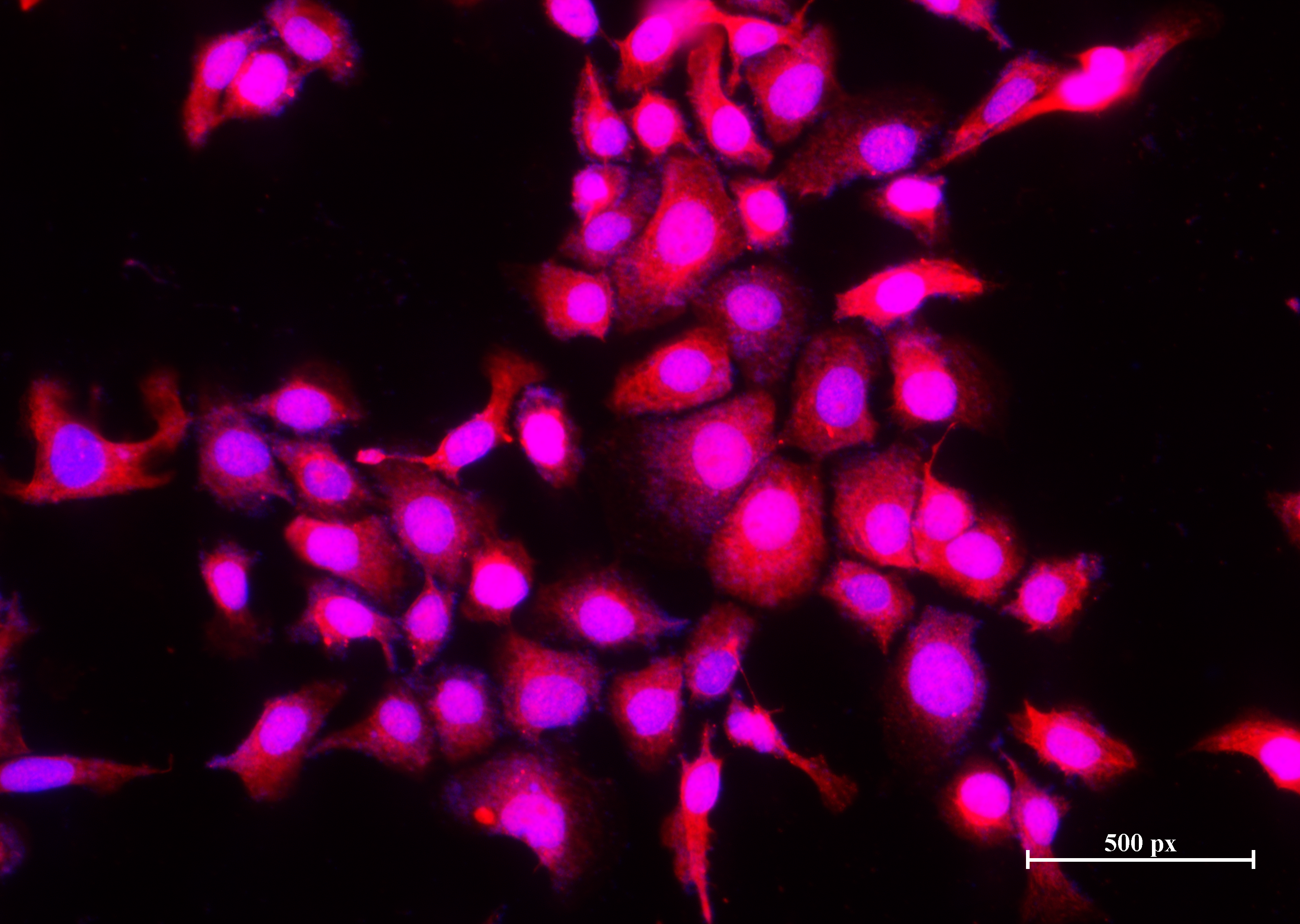

Supplement: Supplementary file 9 [file Data_Sheet_7.ZIP › 12hpi 200X Merged.tif]

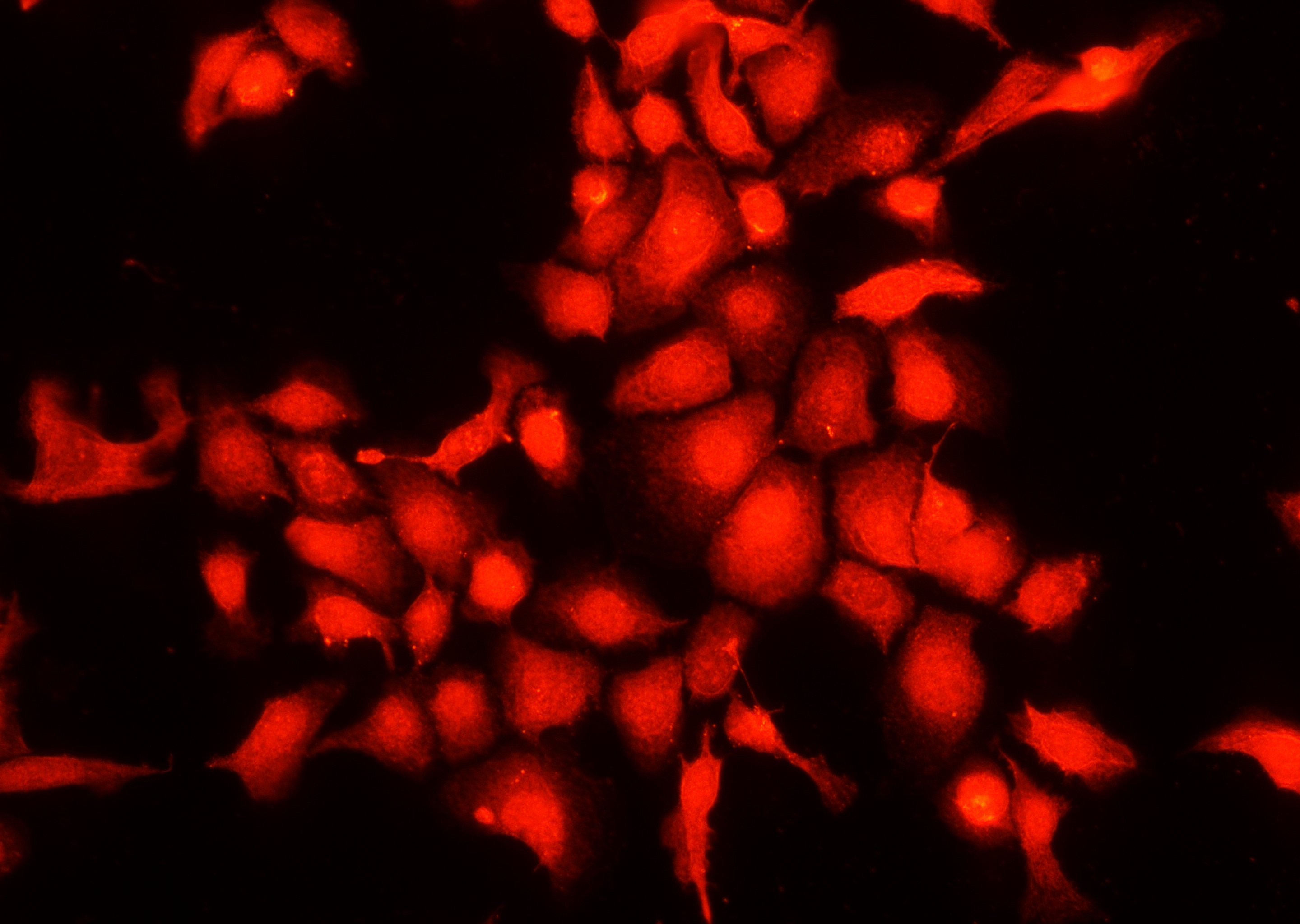

Supplement: Supplementary file 9 [file Data_Sheet_7.ZIP › 12hpi 200X SABC-CY3.tif]
